# Prediction of Complex Human Traits Using the Genomic Best Linear Unbiased Predictor

**DOI:** 10.1371/journal.pgen.1003608

**Published:** 2013-07-11

**Authors:** Gustavo de los Campos, Ana I. Vazquez, Rohan Fernando, Yann C. Klimentidis, Daniel Sorensen

**Affiliations:** 1 Biostatistics Department, University of Alabama at Birmingham, Birmingham, Alabama, United States of America; 2 Animal Science Department, Iowa State University, Ames, Iowa, United States of America; 3 Division of Epidemiology and Biostatistics, The University of Arizona, Tucson, Arizona, United States of America; 4 Department of Molecular Biology and Genetics, Aarhus University, Tjele, Denmark; University of Melbourne, Australia

## Abstract

Despite important advances from Genome Wide Association Studies (GWAS), for most complex human traits and diseases, a sizable proportion of genetic variance remains unexplained and prediction accuracy (PA) is usually low. Evidence suggests that PA can be improved using Whole-Genome Regression (WGR) models where phenotypes are regressed on hundreds of thousands of variants simultaneously. The Genomic Best Linear Unbiased Prediction (G-BLUP, a ridge-regression type method) is a commonly used WGR method and has shown good predictive performance when applied to plant and animal breeding populations. However, breeding and human populations differ greatly in a number of factors that can affect the predictive performance of G-BLUP. Using theory, simulations, and real data analysis, we study the performance of G-BLUP when applied to data from related and unrelated human subjects. Under perfect linkage disequilibrium (LD) between markers and QTL, the prediction R-squared (R^2^) of G-BLUP reaches trait-heritability, asymptotically. However, under imperfect LD between markers and QTL, prediction R^2^ based on G-BLUP has a much lower upper bound. We show that the minimum decrease in prediction accuracy caused by imperfect LD between markers and QTL is given by (1−*b*)^2^, where *b* is the regression of marker-derived genomic relationships on those realized at causal loci. For pairs of related individuals, due to within-family disequilibrium, the patterns of realized genomic similarity are similar across the genome; therefore *b* is close to one inducing small decrease in R^2^. However, with distantly related individuals *b* reaches very low values imposing a very low upper bound on prediction R^2^. Our simulations suggest that for the analysis of data from unrelated individuals, the asymptotic upper bound on R^2^ may be of the order of 20% of the trait heritability. We show how PA can be enhanced with use of variable selection or differential shrinkage of estimates of marker effects.

## Author Summary

Despite great advances in genotyping technologies, the ability to predict complex traits and diseases remains limited. Increasing evidence suggests that many of these traits may be affected by a large number of small-effect genes that are difficult to detect in single-variant association studies. Whole-Genome Regression (WGR) methods can be used to confront this challenge and have exhibited good predictive power when applied to animal and plant breeding populations. WGR is receiving increased attention in the field of human genetics. However, human and breeding populations differ greatly in factors that can affect the performance of WGRs. Using theory, simulation and real data analysis, we study the predictive performance of the Genomic Best Linear Unbiased Predictor (G-BLUP), one of the most commonly used WGR methods. We derive upper bounds for the prediction accuracy of G-BLUP under perfect and imperfect LD between markers and genotypes at causal loci and validate such upper bounds using simulation and real data analysis. Imperfect LD between markers and causal loci can impose a very low upper bound on the prediction accuracy of G-BLUP, especially when data involve unrelated individuals. In this context, we propose and evaluate avenues for improving the predictive performance of G-BLUP.

## Introduction

Many important human traits and diseases are moderately to highly heritable. This, together with advances in genotyping and sequencing technologies, brought the promise of genomic medicine [1]. In the last decade genome-wide association studies (GWAS) have uncovered an unprecedented number of variants significantly associated with important complex human traits and diseases [2]. However in most cases, the combined effects of variants found to be significantly associated with various traits and diseases explain such a small proportion of inter individual differences in genetic risk that the usefulness of genomic information in clinical practice remains limited. In part, this reflects lack of power of standard GWAS to detect phenotype-marker associations for small effect variants [3], [4]. A number of studies have shown that prediction accuracy can be increased by including in the model variants that may not show significant association at the marginal level (e.g., [5]. A few authors [6]–[8] went further and suggested that the analysis and prediction of complex traits may be improved with the use of Whole-Genome Regression methods (WGR; [9]) where phenotypes are regressed on hundreds of thousands of markers concurrently. For instance, using G-BLUP (Genomic Best Linear Unbiased Predictor, one of the most commonly used WGR methods) Yang et al. [7] found that roughly 50% of the genetic variance of human height can be explained by regression on common SNPs. Similar results were confirmed for other complex traits [10].

The ability of a model to predict yet-to-be observed phenotypes (hereinafter referred to as PA, for prediction accuracy) constitutes one of its most important properties from the perspective of its potential use for preventive and personalized medicine. The study by Makowsky et al. [8] assessed PA of G-BLUP and, using family data, reported a cross-validation R^2^ of 0.25. However, the R^2^ ranged from 0.36 for individuals having 3 or more close relatives in the training data set to 0.11 for individuals with no close relatives in the training data set. The result confirms previous findings from the field of animal breeding [11] suggesting important influences of close familial relationships on the PA of G-BLUP methods. This raises an important question: what levels of PA could be expected when G-BLUP is used to predict complex human traits and diseases using data from unrelated individuals?

In this article, using theory, simulation and real data analysis we study the factors that affect the extent of missing heritability and the prediction accuracy of G-BLUP for the analysis of human data. The article is organized as follows. The **methods section** begins with an **overview** of **G-BLUP**. We describe the assumptions that define the model and derive **analytical expressions** that relate genomic relationships to **prediction accuracy** in two scenarios: (a) when the genotypes used for analysis are those at causal loci (hereinafter referred to as analysis under perfect LD between markers and QTL) and (b) under **imperfect linkage disequilibrium** (LD) between the markers used to compute genomic relationships and the genotypes at causal loci. The derivation of the R^2^ formula under perfect LD between markers and QTL follows from standard properties of the multivariate normal density and similar results have been presented before [12], [13]. However, under imperfect LD the model does not hold (because of misspecification of the covariance function) and the standard formulas cannot be used. Based on a few assumptions we derive a closed-form upper bound on prediction R^2^ for the case of imperfect LD. Predictions from the formulas derived in the methods section are validated in **simulated and real data analyses** using **data from related** (Framingham Heart Study [14]) **and nominally unrelated** (a sub-study of GENEVA [15]) **individuals**. In the **Discussion** section the analytical and empirical findings of our research are discussed and put into context and various implications of our results are considered.

## Materials and Methods

Standard quantitative genetic models describe phenotypes 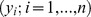 as the sum of a genetic value (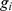) plus an error term 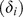; that is 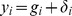. For ease of presentation it is assumed throughout this article that phenotypes and genetic values have null mean (i.e., these are expressed as deviations from the sample mean). Genetic values could be a complex function of the genotypes (*z_i_*) of individual *i* at *q* causal loci, 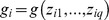 that depends on the genetic architecture of the trait (i.e., the number and exact set of causal loci and the types of interactions among alleles within and between loci, and the distribution of effects). In practice, the genetic architecture of the trait analyzed is unknown and empirical models are built using regressions on marker genotypes. In WGR models [9], phenotypes are regressed on potentially hundreds of thousands of marker covariates 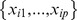 concurrently using a regression function 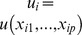 which could be parametric or not. The empirical model becomes: 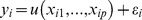, where 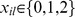 denotes the number of copies of one of the alleles observed on the *i^th^* individual at the *l^th^* marker and 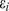 is a model residual that captures the effects of non-genetic factors (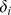) as well as errors 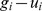 which may emerge either because of model misspecification (e.g., unaccounted interactions) or because of imperfect LD between markers and genotypes at causal loci. In most applications, 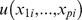 is structured using a parametric linear regression of the form 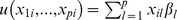 where 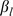 represents the additive effect of the allele coded as one at the *l^th^* marker. Often the number of markers (*p*) vastly exceeds the number of data points (*n*) and implementing these *large-p with small-n* (*p>>n*) regressions requires shrinkage estimation or use of some form of variable selection. Owing to developments in the field of penalized and Bayesian regressions, there is a multiplicity of methods that can be used to implement these *p>>n* regressions [16]. Most of the applications in plant and animal breeding and most of the studies involving human data have used G-BLUP and we therefore focus on this method.

### Genomic Best Linear Unbiased Predictor (G-BLUP)

Genomic BLUP can be motivated in many different ways: as a Ridge Regression (RR, [17]) on marker genotypes, as a Bayesian Gaussian Regression on markers or as a random effects model. A detailed description of this model is given in Supplementary Methods. Here we briefly describe G-BLUP adopting the random effects perspective where phenotypes are viewed as the sum of a random effect representing genomic signal (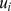) and a model residual (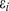),
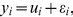
(1)both of which are assumed to follow multivariate normal (MVN) distributions. The vector of genomic values 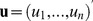 is assumed to follow a MVN distribution with mean equal to zero and variance-covariance matrix proportional to 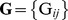, a marker-derived matrix of realized genomic relationships between pairs of individuals (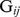). Model residuals, 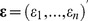 are regarded as independent of ***u*** and assumed to follow IID normal distributions, centered at zero and with variance 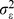. Therefore,
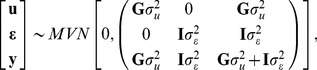
(2)where **I** denotes an identity matrix of dimension *n*. Importantly, the ability of the model described by expressions (1) and (2) to separate signal (**u**) from noise (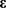) depends completely on how well **G** describes realized genetic relationships at unobserved causal loci.

In empirical analyses, genomic relationships are usually computed using crossproduct terms between genotypes. In such cases, estimates derived from G-BLUP methods are equivalent to those that can be derived by regressing phenotypes on marker genotypes using a linear model, 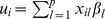, with marker effects treated as IID draws from a normal distribution, 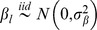. See Supplementary Methods for further details about the equivalence of G-BLUP and some linear regressions on marker covariates.

#### Inferences

Estimation of variance parameters in G-BLUP is possible only when **G** is different from the identity matrix. If that is the case, variance parameters can be estimated from data using Maximum Likelihood, Restricted Maximum Likelihood or Bayesian Methods. The ratio of variance components 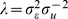 can be viewed as a noise-to-signal ratio and controls the extent of shrinkage of estimates in G-BLUP. Moreover, upon appropriate standardization of **G** (See Supplementary Methods), the ratio
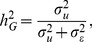
(3)(hereinafter referred as to ‘genomic heritability’) can be regarded as the proportion of variance of phenotypes that is accounted for by regression on the set of markers used to compute **G**. If **G** is computed using genotypes at causal loci 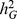 equals the heritability of the trait, denoted as 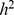. However, when markers are in imperfect LD with genotypes at causal loci, markers may not account for 100% of the variance generated at causal loci and 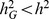. In this case, 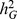 could be regarded as a measure of the proportion of variance at causal loci that can be explained by regression on markers using G-BLUP, in the training sample.

#### Predictions

G-BLUP methods can also be used to predict yet-to-be observed phenotypes. For instance, using a set of *n* phenotypes, denoted as 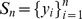, the so called ‘training’ data set (TRN), we may wish to predict the unobserved phenotypic outcome of a new individual in the test or validation data set (TST; hereinafter, and without loss of generality, denoted as 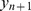). Using standard properties of the MVN distribution we show in the Supplementary Methods (see expression S9.b) that the expected value of 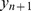 given a TRN sample (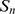) is a weighted sum of the TRN phenotypes; specifically:
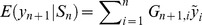
(4)where 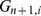 denotes the genomic relationship between individual *n+1* in the TST data set and the *i^th^* individual in the TRN data set, and 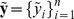 represents a set of ‘*smoothed*’ phenotypes computed by premultiplying the phenotypes of individuals in the TRN data set by matrix 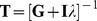. That is: 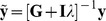, or, in scalar notation, 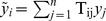 where 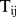 represents the *ij^th^* entry of matrix 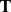, which is proportional to the inverse of the phenotypic (co)variance matrix of observations in the TRN data set.

The weights in this linear score (4) are given by the 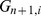
's coefficients that quantify the realized genomic relationships between the individual whose phenotype we wish to predict (typically, from the TST data set) and those individuals available for training. The expected value of the 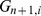
's coefficients equals twice the kinship coefficient between pairs of individuals [11], [18], [19]. For example, in absence of inbreeding and of assortative mating, the expected value of 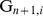 equals 0.5 for parent/offspring or full-sib pairs, or 0.25 for grand-parent/grand-children or half-sib pairs.

### Prediction Accuracy in G-BLUP

The predictive ability of a model is commonly assessed using the variance of prediction errors (or prediction error variance), 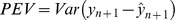, where 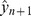 represents a prediction, for instance, 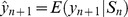. The proportional reduction in phenotypic variance accounted for by predictions (referred to as R^2^ in this article) can be quantified using
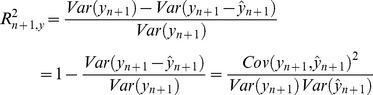
where, 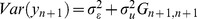 represents the phenotypic variance of individual *n+1*. Below we look at two scenarios: (*i*) prediction accuracy when markers and QTL are in perfect LD and (*ii*) prediction accuracy when markers and QTL are in imperfect LD.

#### Case 1: Prediction accuracy when markers and QTL are in perfect LD

Formulas for PEV and R^2^ for the case when the model holds have been derived elsewhere [12], [13]. It is well known that when the model defined by expression [2] holds prediction R^2^ in validation data sets is given by
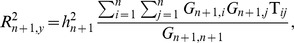
(see the steps leading to expression (S.11) in the Supplementary Methods for a detailed derivation of the above result). The first term in the right-hand side of the above-expression, 
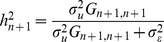
, can be interpreted as an individual-specific heritability; in absence of inbreeding the expected value of this term is 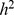. The second term of the right hand side of the above expression represents the R^2^ of prediction of genetic values, 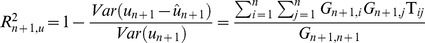
(see eq. (S.11) of the Supplementary Methods). When the model holds, asymptotically, as *n* tends to infinity, 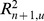 approaches 1; therefore, with perfect LD between markers and QTL, asymptotically, 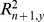 approaches 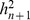.

To get further insight on the role played by TRN-TST relationships, assume for now that the off-diagonal elements of **G** (i.e., all the 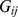 in the TRN data such that 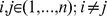) are zero. In this case **T** is diagonal and the R^2^ formula reduces to
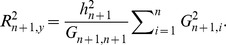



The above expression gives interesting insights into the impact of realized genomic relationships (at causal loci) between TRN and testing (TST) samples on prediction accuracy. When individuals in the TRN data set are genetically independent (i.e., when **G** is diagonal) the individual contribution to each training data point to prediction accuracy of TST phenotypes is proportional to the squared genomic relationship existing between the individual we wish to predict (*n+1*) and the *i^th^* individual in the TRN data set. If a coefficient is small in absolute value (e.g., 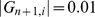) the contribution of the *i^th^* observation to prediction accuracy of the phenotype of individual *n+1* will be minimal. In practice, individuals in the TRN data set will have various degrees of genetic similarity (i.e., **G** will not be diagonal). In this case, the index 
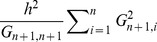
 represents an **upper bound on R^2^**; specifically, under perfect LD between markers and QTL:
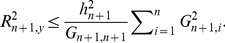
(5)This happens because, all else being equal, the amount of information provided by TRN data is maximized when these data are genetically independent.

#### Case 2: Prediction accuracy when markers and QTL are in imperfect LD

The set of causal loci is typically unknown and in practice, marker genotypes are used to compute genomic relationships. The patterns of realized genomic relationships at different sets of loci (e.g., markers and causal loci) vary across the genome [20]. Because of this, marker-derived genomic relationships may provide a poor description of realized relationships at causal loci, **G**, violating an important assumption of model (2). When the empirical model does not hold, it is not possible to derive closed-form expressions for R^2^. To circumvent this problem, a closed-form expression for an upper bound to R^2^ is obtained instead. The details of the derivation are given in section 2.2 of the Supplementary Methods. Briefly, to arrive at a closed-form upper bound it is assumed that genomic relationships realized at causal loci among pairs of individuals in the TRN data set are known. Therefore, we consider only the impacts of imperfect LD between markers and QTL that occur through misspecification of genetic relationships at causal loci between individuals in the TST and those in the TRN data set.

Prediction equations in GBLUP are given by 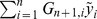. Since by assumption genomic relationships at causal loci among individuals in the TRN data set are known, inferences about the 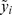
's, i.e., the entries of the vector 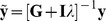, are not affected by imperfect LD between markers and QTL. Let 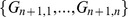 and 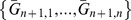 represent the genomic relationships between an individual *(n+1)* in the TST data set and all the *n* individuals in the TRN data set, realized at causal loci and at markers, respectively. Assume that the two sets of genomic relationships can be related according to the following linear model
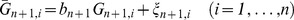
(6)where 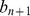 represents the regression of genomic relationships realized at markers 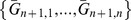 on genetic relationships realized at causal loci 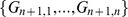 for individual *n+1* and 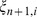, represents a residual term orthogonal to 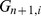. Later in this study the validity of [6] is assessed by computing genomic relationships at disjoint subsets of markers using real genotypes.

A regression similar to (6) was used by [7] to quantify the proportion of unexplained variance (‘missing heritability’) due to imperfect LD. However, the objectives of Yang et al. [7] and ours are different, and consequently the methods used are different. Yang et al. [7] focuses on quantifying effects of imperfect LD on estimates of variance parameters; here we focus on quantifying how misspecification of TRN-TST genomic relationships affects prediction accuracy. Because the focus of the article by Yang and colleagues [7] is on quantifying the effects of imperfect LD on estimates of variance parameters, the regression of realized genomic relationships at subsets of loci is computed using both diagonal and off-diagonal terms of **G**. Here, since we focus on effects of misspecification of TRN-TST relationships we apply the regression of eq. [6] to off-diagonal terms only. These terms, especially those with low expected value (i.e., those involving distantly related pairs), are very sensitive to the effect of imperfect LD [20].

**Figure 1:**
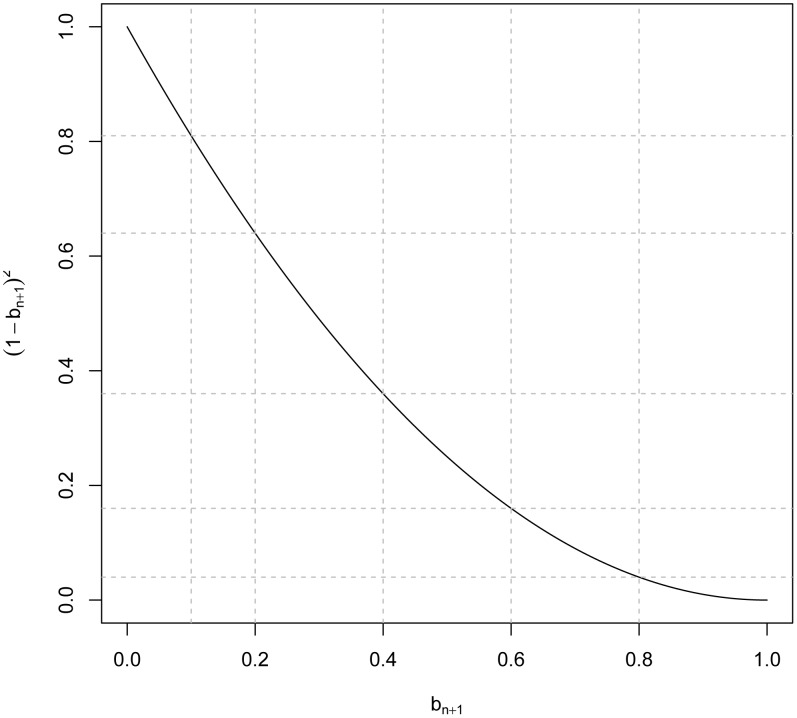
Minimum R^2^ reduction factor, 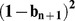, due to imperfect linkage disequilibrium between markers and QTL versus values of the regression of genomic relationships realized at markers and at causal loci (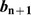).

Using (2) and (6) it can be shown (see eq. (S.14) of the Supplementary Methods) that the R^2^ that can be attained using markers that are in imperfect LD with genotypes at causal loci (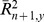) satisfies the following inequality:
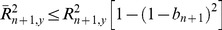
(7)The right hand side of the above expression has two terms: the first one, 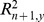, is the R^2^ that could be obtained with the same TRN sample if markers were in perfect LD with the QTL. **The second term**, 
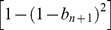
, quantifies the reduction in prediction R^2^ that occurs due to imperfect LD between markers and QTL. **The term** 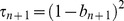 **represents a minimum proportional reduction on prediction R^2^ due to imperfect LD between markers and QTL.** This is so because the derivation of (7) only considered the impact of imperfect LD between markers and QTL on the computation of the genomic relationships between the individuals in the TST data set and those used for TRN. In practice, the relationships among individuals in the TRN data set are also estimated from markers that are in imperfect LD between markers and QTL. This will also have a detrimental effect on PA, which is not accounted for by the right hand side of expression (7). Asymptotically and in absence of inbreeding 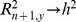; therefore, with large samples 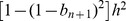 becomes an upper-bound to prediction R^2^ under imperfect LD. Figure 1 displays values of the minimum reduction factor, 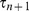, versus 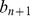. In practice the set of causal loci is unknown and computing 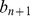 is not feasible. However 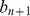 can be estimated by computing genomic relationships at different sets of loci and subsequently regressing the realized genomic relationships at different sets of loci on one another. An example of this approach is offered in the next section.

The principles used to derive expression (7) can also be applied when predictions are computed from pedigree information as opposed to markers. In this case 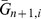 should be replaced by the pedigree based relationships. That is: 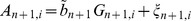 where now 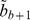 represents the regression of pedigree derived relationships, 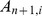, on realized genetic relationships at causal loci. In this case, departure of 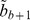 from 1 reflects a deviation between the pedigree based relationship and the realized genetic relationship at causal loci.

### Simulation and Real Data Analysis

To obtain further insight on the impacts of imperfect LD between markers and QTL on the proportion of missing heritability and on PA, a simulation study and real data analysis were performed using data sets from related and from unrelated individuals.

#### Data

We used two publically available data sets: one involving family data (the Framingham Heart Study, hereinafter denoted as FHS, [14]) and one (GENEVA, hereinafter denoted as GEN, [15]) with nominally unrelated individuals– defined based on available pedigree information. The data contained in GEN was collected by the Gene-Environment Association Studies consortia (https://www.genevastudy.org/). Here, we used the type-2 diabetes case-control data sets which were drawn from the Nurses' Health Study and the Health Professionals Follow-up study [15].

#### Ethics statement

The FHS data set required IRB approval and this was done at the IRB of the University of Alabama at Birmingham (Protocol # X100712003). GEN did not qualify as human subject data.

Individuals in the FHS were genotyped with a 500 K SNP platform (K = 1,000) and those in GEN were genotyped with a 1,000 K SNP platform. We identified a set of 400 K SNPs which were available both in GEN and FHS and passed quality control criteria which consisted of removing markers with more than 10% of uncalled genotypes and with minor allele frequency (MAF) smaller than 0.5%. These 400 K SNPs were used for simulations and for data analysis, as described next. In both data sets individuals with unknown height, age or sex were removed in order to allow comparison with analysis of the real data presented in the next section.

In GEN, only nominally unrelated individuals of Caucasian origin with less than 5% of missing genotypes were included. This left 5,854 individuals out of which 5,800 were randomly sampled for the analysis. In FHS individuals with no data from relatives and subjects with more than 5% of uncalled genotypes were removed. The 7,865 individuals that passed these criteria were ranked using an index consisting of the sum of squares of the additive relationships (computed from pedigree) of each individual with the rest of the data set 
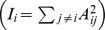
. And based on this index, the highest 5,800 ranking individuals were kept for analysis. With this strategy, the sample size of both data sets was matched and at the same time, the degree of relationships existing among individuals in FHS was maximized. This generated a maximally contrasting scenario between the two data sets.

#### Simulation

The simulation study incorporates the observed genotypes from the FHS and GEN data sets as follows. From the available 400 K SNPs, 300 K were randomly sampled and designated as markers (hereinafter genotypes at these loci are denoted as 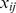, *j = 1,…,300* K). Causal loci (*p* = 5,000, denoted as 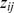) were sampled from the remaining 100 K SNPs using two strategies: (a) completely at random (RAND, by assigning equal sampling probability, 1×10^−5^, to all markers) or (b) at random but sampling an excess of SNPs with low MAF (Low-MAF). Here we assigned sampling probability of 0 to markers with MAF greater than 0.15, 1.76×10^−5^ probability to markers with MAF between 0.05 and 0.15 and three times higher probability, 5.28×10^−5^, to markers with MAF smaller or equal to 0.05. These sampling probabilities were defined based on MAF computed using the average MAF observed in FHS and GEN. In the RAND scenario, markers and genotypes at causal loci were sampled from the same distribution; on the other hand, in the Low-MAF scenario markers and genotypes at causal loci have different distribution of allele frequencies.

The effects of causal loci 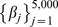 and model residuals 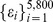 were sampled from independent normal densities, the residual variance (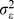) was set to 0.2 and the variance of causal loci effects (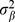) was set to: 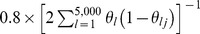 where the 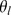
's correspond to observed allele frequencies calculated pooling the genotypes in FHM and GEN. Phenotypes were finally generated using a linear model of the form: 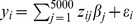. Under linkage equilibrium, this results in a phenotypic variance and heritability equal to 1 and 0.8, respectively. A total of 30 Monte Carlo (MC) replicates were generated. The assignment of markers into causal and tag SNPs was kept fixed across MC replicates; therefore, what varied across replicates were causal loci effects and error terms. Note that the assignment of SNPs to sets of markers and of causal loci, as well as the marker effects and error terms used in the simulations were the same in FHS and GEN; therefore, the only difference between the two data sets in the simulation study was that one (FHS) consisted of related individuals and the other (GEN) of unrelated subjects.

#### Analysis of simulated data

The simulated data were analyzed using a G-BLUP model with genomic relationships computed either using genotypes at causal loci (*G*) or using markers (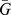) and according to the following expressions:
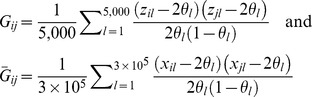



There are several ways of computing genomic relationships [7], [21]–[24]; here, we have adopted one of the conventions which consist of centering (by subtracting the mean, 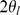) and standardizing, by dividing by the variance 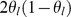, of each marker before computing genomic relationships. The allelic frequencies used to center and standardize genotypes were computed by pooling the genotypes in the FHS and GEN data sets. Centering has no effects either on estimation or predictions [25]. On the other hand, standardization does have an effect. When marker genotypes are standardized all markers make an equal contribution to the computation of relationships. An advantage of centering and standardizing is that the expected value of the resulting genomic relationship matrix is the numerator relationship matrix; therefore, deviations of the observed genomic relationship matrix relative to its expected value can be attributed to the sampling of alleles at meiosis.

In the analysis of the simulated data a Bayesian model with uninformative priors for variance parameters was used to estimate variance parameters and to predict phenotypes and genetic values. Details of the model used for analysis as well as specifics of the MCMC implementation are given in Supplementary Methods.

#### Estimation of prediction accuracy in simulated data

For each of the 30 MC replicates, we used data from 5,300 individuals to train the models (TRN) and data from 500 individuals for testing (TST). Phenotypes of individuals assigned to TST were regarded as missing and prediction accuracy was assessed by means of R^2^ between predicted and observed phenotypes in the TST data sets, estimated by the squared of Pearson's product-moment correlation. The assignment of individuals to TRN/TST data sets was completely at random in GEN. In FHS we designed a sampling strategy that guarantees that prediction used only ancestors and nominally unrelated individuals. Specifically, TST data sets were drawn from the most recent cohort, and the algorithm used to construct the TRN data set avoided contemporaneous relatives between individuals in the TRN and TST data sets.

#### Real data analysis

In the real data analysis height is used as model trait and we report estimates of variance parameters and assess PA in FHS and GEN. The models for the analysis were similar to those used in the simulation (see below for details) but alternatives that incorporate results from previous GWAS into the whole-genome regression approach are also considered. In both data sets height was preadjusted with estimates of effects of age and sex (estimated within each data set). Estimates of variance components were derived by fitting models to each of the full data sets (N = 5,800, in both cases) and to the combined data sets (N = 11,600). PA was assessed for FHS and GEN using the same 30 TST data sets used in the simulation, each containing 500 individuals.

For assessing PA, the training was done within data set (N-TRN = 5,300 in each partition) or using a combined data set. For the combined data set analyses, when testing was carried out in GEN, the TRN data set included the 5,300 individuals from GEN (those not used for TST in each partition) plus 5,800 from FHS. Similarly, when testing in FHS, the TRN data set included 5,300 individuals from FHS and 5,800 from GEN.

#### Models used in the real data analysis

The baseline model was a G-BLUP using all available markers (p = 400 K SNPs). As shown in the next section, results from the simulation study suggest that imperfect LD between genotypes at markers and those at causal loci can have dramatic impacts on PA, especially when data involve unrelated individuals. In practice, the set of causal loci is unknown; however, it is possible to use information from existing GWAS to either select or weight the markers included in the analysis. Therefore, we also evaluated G-BLUP models using a subset of markers selected on the basis of their association p-values for human height published by the GIANT consortium [5]. To this end, SNPs were ranked according to their p-values (from smallest to largest) and then G-BLUP type models were implemented using the top-*t* markers only, with *t* = 100, 250, 500, 1 K, 2.5 K, 5 K, 10 K, 20 K, 50 K and 100 K.

As an alternative to this variable selection approach we evaluated the use of genomic matrices computed by weighting all markers differentially [26], in this case according to the evidence of association obtained from the GIANT study. Specifically, in this weighted-G-BLUP (hereinafter wG-BLUP) genomic relationships were computed as 
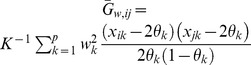
, where 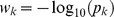, 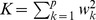, and 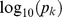 is the base-10 logarithm of the SNP association p-value reported by the GIANT consortium [5]. These p-values were derived using a sample size of the order of 133,000 records. Although both FHM and GEN were part of this study, in practice, in each replicate we are validating using 500 individuals; arguably, the influence of these data points on the derivation of the p-values derived by the GIANT consortium is negligible. The fourth and final set of results was obtained by predicting phenotypes using pedigree information only. This approach is denoted P-BLUP (pedigree BLUP) and was applied only to FHS.

## Results

Results from the simulation study are reported first; this is followed by the results of the real data analysis.

### Results from the Simulation

Table 1 shows the distribution of allele frequencies (computed among the 5,800 individuals used for analysis in each of the data sets) by set of markers and data set. The distribution of allele frequencies observed in the FHS and GEN was very similar, with a correlation of MAF between the two data sets of 0.997. The distribution of minor allele frequencies of subsets of randomly chosen markers (either those designated as non-causal loci or those designated as causal loci in the RAND scenario) was very similar, with more than 65% of the markers having a MAF greater than 0.15. On the other hand, as a consequence of the sampling scheme used, in the Low-MAF scenario, the distribution of allele frequencies at causal loci had an over representation of low MAF loci. We also computed the squared correlation of genotypes of adjacent markers at various lags (in this case defined as the number of markers in the interval in the map), from lag 1 to lag 100 in FHS and GEN. For the set of SNP used in this study (400 K) the average inter marker distance was 7.2 kb. Plots of the patterns of association between genotypes at adjacent markers are given in the Supplementary Data (see figures S1 and S2). Although, for some pair of markers, the squared correlations in FHS and GEN were different; however, the overall patterns (e.g., the average squared-correlation at lag 1, 2,…, 100, or percentiles of the squared correlations at various lags) were identical in both data sets.

**Table 1:** Percentage of loci by minor allele frequency (MAF), scenario and data set.

Type of loci	Scenario	Data set	Minor Allele Frequency				
			<3%	3%–5%	5%–10%	10%–15%	>15%
Tag		FHS	.061	.049	.119	.116	.654
Tag		GEN	.065	.049	.119	.115	.652
Causal	RAND	FHS	.063	.047	.117	.123	.651
Causal	RAND	GEN	.066	.048	.117	.117	.651
Causal	Low-MAF	FHS	.310	.233	.239	.207	.011
Causal	Low-MAF	GEN	.321	.237	.231	.201	.010

FHS = Framingham Heart Study, GEN = GENEVA, RAND: in this scenario causal and marker loci were drawn from the same distribution, Low-MAF: in this scenario marker loci were drawn at random and causal loci were drawn over-sampling loci with extreme minor-allele frequency (MAF). In the Low-MAF scenario, the sampling of causal loci was done using average MAF of the FHS and GEN data sets. Although MAF were very similar across data sets, these were not exactly equal, and this explains why in the Low-MAF roughly 1% of the causal loci had MAF (within-data set) greater than 15%.

The eigenvalue decomposition of the marker-derived genomic relationship matrices revealed that the cumulative variance explained by the 1^st^ 5 eigenvalues were 0.35, 0.51, 0.64, 0.78 and 0.90% in FHM and 0.35, 0.51, 0.61, 0.69, and 0.77% in GEN, respectively. Ordinary least squares regression of adjusted height on the 1^st^ PC explained a proportion of the variance (in the training sample) equal to 4% in FHM and to 2% in GEN. Therefore, although both data sets exhibit some extent of population stratification, the proportion of variance of genotypes explained by high order principal components was low.

Estimates of 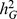 and of prediction R^2^, averaged across 30 MC replicates are displayed in Table 2. Results by MC replicate are provided in Tables S1, S2, S3, S4, S5 of the Supplementary Data.

**Table 2:** Estimates (estimated standard errors) of proportion of variance explained (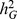) and of prediction R-squared of phenotypes in validation datasets, *R^2^* (TST).

Scenario	Genetic Information Used to Compute Relationships	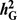 (1)		*R^2^* (TST)(2)	
		FHS	GEN	FHS	GEN
RAND	Causal Loci	0.775	0.773	0.545	0.517
		(0.009)	(0.010)	(0.040)	(0.031)
	Markers	0.774	0.737	0.263	0.071
		(0.018)	(0.040)	(0.048)	(0.023)
	Pedigree	0.764	—	0.223	—
		(0.020)		(0.047)	
Low-MAF	Causal Loci	0.777	0.775	0.551	0.536
		(0.007)	(0.008)	(0.026)	(0.026)
	Markers	0.748	0.573	0.240	0.049
		(0.018)	(0.058)	(0.029)	(0.019)
	Pedigree	0.755	—	0.224	—
		(0.023)		(0.033)	

FHS = Framingham Heart Study, GEN = GENEVA, RAND: in this scenario causal and marker loci were drawn from the same distribution, Low-MAF: in this scenario marker loci were drawn at random and causal loci were drawn over-sampling loci with low minor allele frequency, TST = Testing data set.

(1) : average (over 30 MC replicates) estimated posterior mean of the ratio of genomic variance over the sum of genomic and residual variance;

(2) : average prediction R^2^ (phenotypes) over 30 training (N = 5,300)-testing (N = 500) partitions.

#### Proportion of variance explained

The estimates (± estimated standard error) 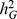 obtained when realized genetic relationships at causal loci were used were very close to 0.8 in FHS (0.78±0.01) and GEN (0.77±0.01). This holds for both sampling scenarios, RAND and Low-MAF. Therefore, as one would expect, and regardless of whether data are from related or unrelated individuals, when genotypes at markers and those at causal loci are in perfect LD, the model holds and there is no missing heritability. On the other hand, when genotypes at markers and those at causal loci are in imperfect LD, in FHS the estimates of 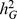 were 0.77 (±0.018, RAND scenario) and 0.75 (±0.018, Low-MAF scenario) and those from GEN were 0.74 (±0.040) and 0.57 (±0.58), for the RAND and Low-MAF scenarios, respectively. Therefore, with family data and imperfect LD we either did not observe missing heritability (RAND scenario) or observed a very small proportion of missing heritability, roughly 3% computed as 100×(1−0.748/0.777), when marker and causal loci were drawn from different distributions (Low-MAF scenario). However, with unrelated individuals (GEN) we either observed a small proportion of missing heritability, roughly 4% (computed as 100×[1−0.737/0.773) in the RAND scenario, or a great deal of missing heritability, roughly 26% (computed as 100×[1−0.573/0.775]), in the Low-MAF scenario. These results are consistent with previous analyses of human height using related [7], [8] individuals and provide support to the conjecture offered by Yang et al. [7] that the extent of missing heritability observed with unrelated individuals may be due to imperfect LD between markers and QTL, exacerbated by the fact that marker and causal loci may have different distributions of allele frequency.

For the FHS we also fitted a pedigree-based model to the simulated data and the estimates of proportion of variance explained with pedigrees (0.764 and 0.755 in the RAND and Low-MAF scenarios, respectively) were very similar, only slightly lower, but not significantly different based on the MC SEs, to those obtained when genotypes at markers were used.

#### Prediction accuracy

When genotypes at marker and causal loci are in perfect LD, the R^2^ between predicted and observed phenotypes in TST data sets (averaged across 30 MC replicates) ranged from 0.517–0.551, with very minor differences across data sets and scenarios. The R^2^ for prediction of genetic values (not presented in the Table 2) are given in the Supplementary Data (see Tables S4 and S5).

The PA attained when marker genotypes were used to compute genomic relationships was much lower than that achieved using genotypes at causal loci. Reduction in R^2^ due to imperfect LD between markers and QTL ranged from 52% (for FHS in the RAND scenario, computed as 100×[1−0.263/0.545]) to 91% (for GEN in the Low-MAF scenario, computed as 100×[1−0.049/0.536]). In both data sets the reduction in R^2^ was higher in the Low-MAF scenario than in the RAND scenario; however, the reduction in R^2^ was orders of magnitude different in FHS and GEN, regardless of the simulation scenario.

Importantly, in many cases, the value of the estimated 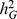 did not provide a good indication of what one would expect for prediction R^2^. For instance, in FHS the use of markers, as opposed to causal loci, did not induce a great extent of missing heritability, but the PA attained with markers was less than 50% of that attained when genotypes at causal loci were used. Another example can be seen in GEN; here, in the RAND scenario when markers were used we observed only a small extent of missing heritability (the estimated value was 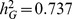), but the reduction in R^2^ due to use of markers that were in imperfect LD with causal loci was dramatic (86% computed as 100×[1−0.071/0.517]).

Finally, in FHS the prediction accuracy of the pedigree model (R^2^ 0.224) was, as one would expect, lower than that of the marker-based model (R^2^ 0.263). Relative to the pedigree model, using markers leads to a gain in PA in the R^2^ (correlation) scale of 17.9% (8.6%), computed as 100×[0.263/0.223–1] 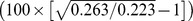. These gains in PA are smaller than those reported in plant and animal breeding populations where LD spans over long regions. For instance, for prediction of estimated breeding values [27] reported an average (over 20 traits) gain in predictive correlation of 20%.

According to expression (7) of section 2, imperfect LD between markers and QTL results in a minimum reduction in prediction R^2^ of 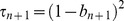. These regression coefficients 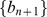
were estimated for each of the 500 individuals in each of the 30 TST data sets used in the simulations. Results (averaged across individuals and MC replicates) are given in Table 3. The average regression coefficient in FHS was much higher than in GEN, and the regression coefficient was lower in the scenario where marker and causal loci were drawn from different distributions. Consequently, according to (7), we anticipate much higher minimum reduction factor in GEN than in FHS and in Low-MAF than in RAND, and this is consistent with the results reported in Tables 2 and 3.

**Table 3:** Average (over individuals in TST data sets) regression coefficient (*b_n+1_*, see eq. 4) between realized genomic relationships at markers and those realized at causal loci, corresponding minimum reduction factor in prediction R^2^ (see eq. 7) and observed reduction factor in prediction R-squared.

Data set	Information used to compute relationships	Simulation Scenario	Regression Coefficient (*b_n+1_*)^1^	Reduction Factor in R-squared	
				Minimum^2^ (*τ_n_*_+1_)	Observed^3^
Framingham	Pedigree	Random	0.295	50%	59%
		Low-MAF	0.285	51%	60%
	Markers	Random	0.371	40%	52%
		Low-MAF	0.334	44%	56%
GENEVA	Markers	Random	0.127	76%	86%
		Low-MAF	0.089	83%	91%

Low-MAF: scenario where causal loci were over-sampled among loci with low minor allele frequency. Random: scenario where markers and causal loci were sampled from the same distribution.

1 : For each individual in the testing (TST) data sets we computed the regression of marker or pedigree derived relationships on genomic relationships computed at causal loci, 
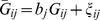
 and 
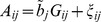
, respectively, where *j* (*j = n+1,n+2,…*) indexes individuals in the testing data set and *i* (*i = 1,…,n*) indexes individuals in the training (TRN) data sets. The TRN-TST partitions used were those used in the simulation. Results in the table are averages across individuals in TST data sets.

2 : Upper bound calculated using expression (7).

3 : Reduction in prediction R^2^ observed when data was analyzed using markers relative to that obtained when data was analyzed using genotypes at causal loci (see Table 2).

The validity of inequality (7) can be evaluated by comparing the minimum reduction factor in prediction R^2^, 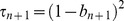, given in the 5^th^ column of Table 3, with the observed reduction factors, computed using results presented in Table 2; these are given in the last column in Table 3. In all scenarios/data sets the minimum reduction factor under predicts the observed reduction in R^2^, as expected from (7). The order of magnitude of 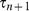 and of the observed reduction in prediction R^2^ are similar, with a difference between the two of roughly 10 percentage units.

**Figure 2:**
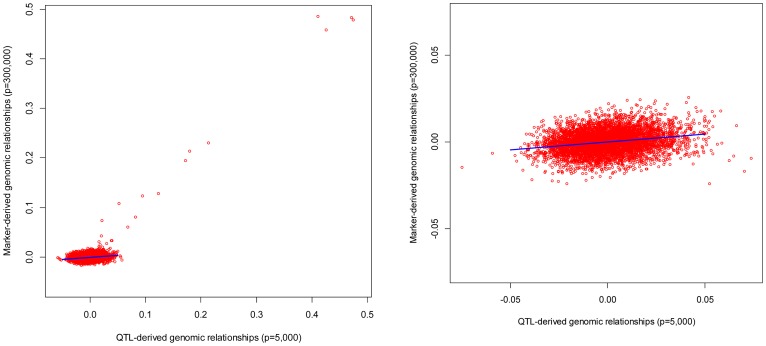
Genomic relationships realized at markers (vertical axis) versus those realized at causal loci (horizontal axis). The plot displays realized relationships between one individual in TST and all the other individuals in TRN for GEN (right panel) and FHS (left panel). Genomic relationships computed using markers are given in the vertical axis and those computed using genotypes at causal loci are in the horizontal coordinate.

To get further insight, and to check the validity of the linear relationship postulated by eq. (6), off-diagonal elements of genomic relationship matrices computed from markers were plotted against those realized at causal loci for individuals in FHS and GEN. The patterns observed were relatively consistent across individuals: (a) overall, the linear pattern postulated by eq. (6) seems to hold well in GEN and to some extent in FHS (see Figure 2), (b) large realized genomic relationship coefficients (e.g., genomic relationships between parent-offspring or, more in general, those greater than 0.1) were very similar at markers and at causal loci; therefore, for the regression coefficient 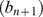 was very close to one; however, (c) the same regression was much lower (of the order of 0.1) for nominally unrelated individuals. This is illustrated in Figure 2 that discloses the realized relationships (at markers in the y-axis and at causal loci in the x-axis) between one individual in TST and all the other individuals in TRN for GEN (right panel) and FHS (left panel). In the case of the right panel (GEN) since the individual is nominally unrelated with all the individuals in the TRN dataset, the regression of the genomic relationships realized at markers on those realized at casual loci is very low (of the order of 0.1). On the other hand, the plot in the left (FHM) shows two contrasting situations: for the small coefficients (i.e., those describing relationships between nominally unrelated individuals) the regression is as flat as in the case of GEN; however the regression approaches one as the coefficients become larger.

The effect of the degree of familial relationships on the regression between genomic relationships realized at markers and at causal loci is further illustrated in Table 4. The table displays estimates of regression coefficients computed for individuals in the TST data that are either related or nominally unrelated to those in the TRN data. As one could anticipate from Figure 2, the estimated regression coefficients were low (of the order of 0.1) for pairs of unrelated individuals both in FHS and in GEN. On the other hand, for related individuals, the estimated regression coefficients were close to one, suggesting that for these pairs of individuals, marker derived genomic relationships provide a very good description of the realized genetic relationships at causal loci.

**Table 4:** Regression coefficient (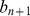, see expression 6) between realized genomic relationships at markers and those realized at causal loci, by data set, type of relationship and simulation scenario.

Data set	Framingham				GENEVA	
Relationships^1^	Related		Unrelated		Unrelated	
Scenario	RAND	Low-MAF	RAND	Low-MAF	RAND	Low-MAF
Average	0.992	0.998	0.119	0.078	0.127	0.089
q_5%_	0.898	0.827	0.085	0.048	0.087	0.051
q_95%_	1.083	1.174	0.184	0.130	0.329	0.269

1 : Relationship between the individual whose phenotype is predicted and those used for model training; coefficients, 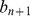, were estimated for each individual in training datasets. q_5%_ and q_95%_ represent the 5% and 95% empirical percentiles of the estimated regression coefficients.

### Results from the Real Data Analysis

Table 5 gives estimated posterior means of 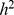 for the pedigree-model (P-BLUP, applied to FHS only), and of 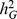 for G-BLUP and wG-BLUP fitted to the full and combined data sets. The estimate of 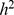 for the P-BLUP model in FHS was 0.857; this value is within the range, slightly higher, of what is generally considered the heritability of human stature (i.e., 0.8). The estimate of 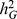 in FHS with G-BLUP was slightly smaller (0.837). Both results are in agreement with previous reports for this trait and data set (e.g., Makowsky et al. [8]) as well as with the simulation study presented in this article, with one small difference: in the simulation study the estimate of 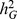 from marker based G-BLUP was slightly higher than that of P-BLUP, while in the real data analysis the opposite happened. One possible explanation is that in the real data analysis P-BLUP captured some non-additive genetic effects and/or some components of permanent environment that are not captured by G-BLUP. Finally, in FHS, the estimated 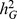 derived using wG-BLUP was similar, albeit slightly lower, than with G-BLUP (0.814). In short, regardless of the method (P-BLUP, G-BLUP or wG-BLUP) no missing heritability is observed in the analysis of family data.

**Table 5:** Estimates of proportion of variance accounted for by regression on pedigree or regression on markers by training data set and analysis method (estimated posterior standard deviaton).

Data set	Pedigree	G-BLUP	wG-BLUP
Framingham (N = 5,800)	0.857	0.837	0.814
	(0.016)	(0.016)	(0.013)
GENEVA (N = 5,800)	—	0.374	0.268
		(0.049)	(0.026)
Framingham+GENEVA (N = 11,600)	—	0.721	0.632
		(0.016)	(0.015)

wG-BLUP uses all SNPs (p = 400 K), but the contribution of each SNP to the genomic relationship matrix was weighted using 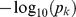 as weight, where 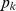 is the SNP associated p-value reported by the GIANT consortium [5].

On the other hand, the analysis of data from unrelated individuals (GEN) exhibited a great extent of missing heritability (roughly 53% for G-BLUP, computed as 100×[1−0.374/0.80]) both for G-BLUP and even greater for wG-BLUP. These results are also in agreement with previous reports for the trait (e.g., [7]) and with the trend observed in the simulation study in scenario Low-MAF. However, the extent of missing heritability was higher than what was observed in the simulation, perhaps suggesting that the levels of imperfect LD between genotypes at markers and those at causal loci affecting human height are even more extreme that those present in the simulation.

#### Prediction accuracy

Table 6 shows estimates of prediction accuracy by model and data set. Estimates of phenotype prediction R^2^ within FHS obtained with P-BLUP and G-BLUP were of the order of 0.28. These results are similar to what was observed for this data set in the simulation, with two main differences: (a) the values of R^2^ obtained in the simulation were 10–20% lower than those in the real data and (b) in the simulation, G-BLUP outperformed P-BLUP but in the real data analysis the opposite happened. This observation is consistent with the conjecture that P-BLUP may be capturing environmental and non-additive effects that are not captured by G-BLUP. The PA of G-BLUP in GEN was very poor (R^2^ of 3.1% when training with GEN only) and this is in agreement with the simulation results. The PA of wG-BLUP was higher than that of G-BLUP in FHS (11% gain in R^2^, calculated as 100×[0.311/0.280–1]) and substantially higher in GEN (R^2^ = 0.086 of wG-BLUP was almost 3 times higher than that of G-BLUP). Combining FHS and GEN for training was beneficial for prediction of GEN but not for prediction of FHS. With use of a TRN data set that included FHS and GEN and with wG-BLUP we attained a prediction R^2^ of 11% with unrelated individuals. Interestingly, in the case of GEN wG-BLUP leads to higher proportion of missing heritability and to higher prediction accuracy, stressing again that there is no direct relationship between estimates of 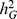 and prediction R^2^.

**Table 6:** Prediction R-squared evaluated in testing data sets (average over 30 randomly drawn testing data sets, each having 500 individuals) by training and validation data sets and model.

Training data sets		Testing data sets		Pedigree-BLUP	G-BLUP	wG-BLUP
N-FHS	N-GEN	N-FHS	N-GEN			
5,300	—	500	—	0.284	0.281	0.311
				(0.048)	(0.051)	(0.037)
5,300	5,800	500	—		0.273	0.290
					(0.048)	(0.036)
—	5,300	—	500		0.031	0.086
					(0.013)	(0.020)
5,800	5,300	—	500		0.036	0.110
					(0.015)	(0.027)

N-FHS = Number of records from Framingham, N-GEN = Number of records from GENEVA. G-BLUP uses 400 K SNPs, wG-BLUP uses 400 K SNPs, but the contribution of each SNP to the genomic relationship matrix was weighted using 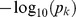 as weight, where 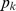 is the SNP associated p-value reported by [5].

**Figure 3:**
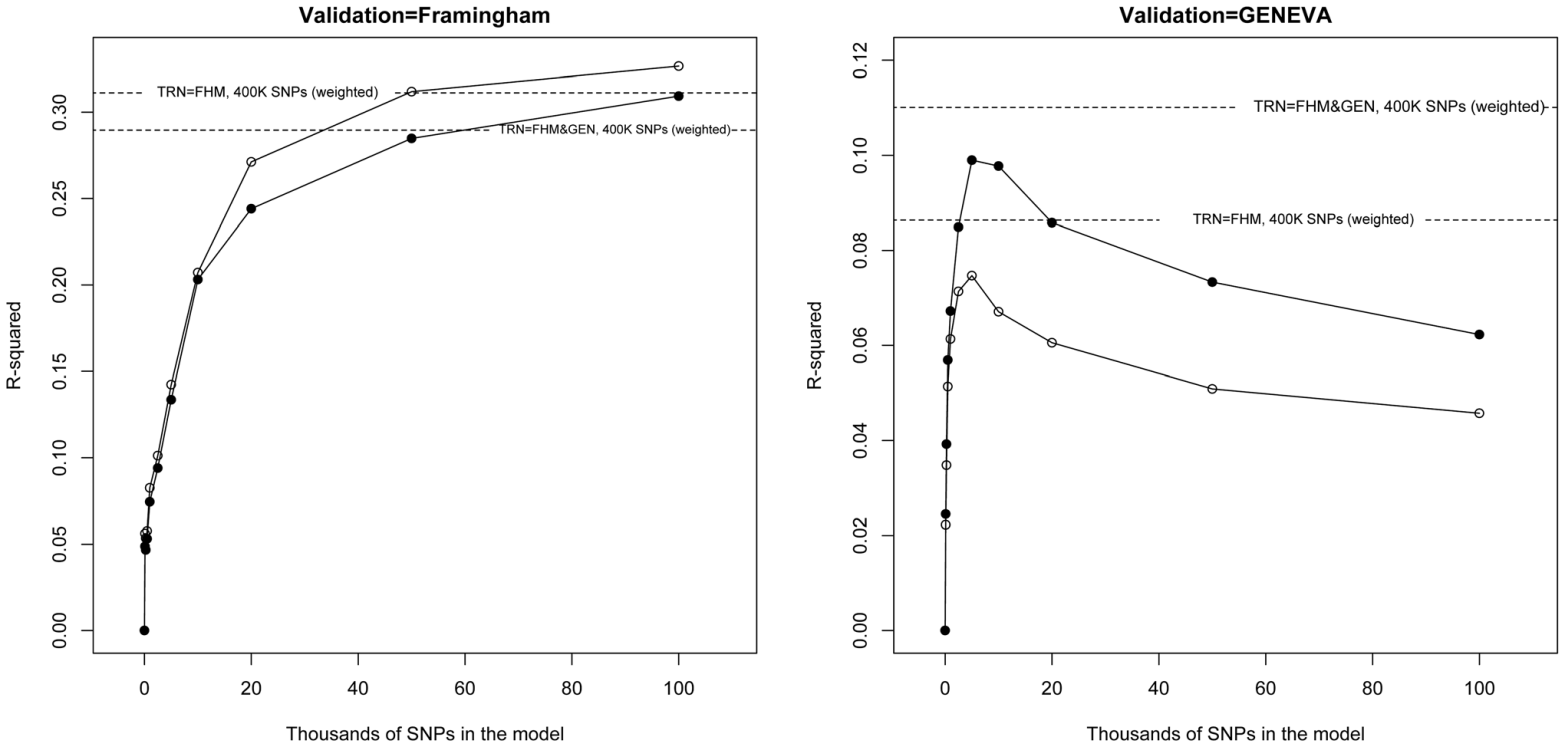
Prediction R^2^ (vertical axis) versus thousands of markers (selected based on p-values from the GWAS of the GIANT consortium, [5]) included in the model (horizontal axis) by validation data set (FHM in the left panel, GEN in the right panel) and training data set (line with dots training with FHM and GEN combined, line with circles, training-testing within each study). Dotted horizontal lines give the prediction R^2^ obtained when all markers (p = 400,000) were used.

The above results indicate that the use of differential weights in the computation of genomic relationships may be beneficial, especially with data from unrelated individuals. Another alternative is to use p-values from GWAS to select predictors. Figure 3 gives prediction R^2^ obtained with FHS and GEN using subsets of markers selected on the basis of the associated p-values reported by the GIANT consortium [5]. In FHS, R^2^ increased monotonically with marker density in the range 0–100 K SNPs, suggesting no benefit of pre-selecting markers within that range. In this data set, R^2^ with 400 K SNPs was only slightly lower than that obtained using the top 100 K SNPs; therefore, we conclude that there is little benefit of performing variable selection when family data are used. On the other hand, for GEN, benefits of pre-selection of markers are clearly observed: in this case R^2^ increases sharply up to 5 K SNPs, achieving a prediction R^2^ substantially higher than the G-BLUP with 400 K SNPs (7.5% relative to 3.1% or 9.9% relative to 3.6% in the analyses with training using GEN or GEN combined with FHS, respectively), and decreases thereafter with higher marker density. However, wG-BLUP gave higher prediction accuracy than the use of 5 K selected SNPs (R^2^ 8.6% and 11% in the case of the analysis using GEN or GEN and FHM for training) suggesting that perhaps the use of ‘smooth weights’ may be better than the use of 0/1 weights which are implicitly used when markers are pre-selected. We note again that the estimates of genomic heritability did not follow the same patterns than those of R^2^, for instance the analysis with the top 5 K SNPs yielded smaller genomic heritability but higher prediction accuracy than the analysis with 400 K SNPs.

## Discussion

In recent years GWAS have uncovered unprecedented numbers of variants associated with many important complex human traits and diseases. However in most cases the joint effects of variants detected so far explain only a small proportion of the genetic variance of those traits, a problem referred to as the missing heritability of complex traits [3], [4]. G-BLUP was first used in human genetic studies partly to address this issue in the study of Yang and co-authors in 2010 [7]. This study showed that inclusion of all available marker information in a joint analysis resulted in a marked increase of the proportion of variance explained, recovering part of the missing heritability. However, the ability of a model to predict yet-to-be observed phenotypes can be markedly different from the proportion of variance accounted for in a training data set, as measured by estimates of 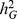. Previous studies [8] suggest that the ability of G-BLUP to predict unobserved phenotypes of individuals that are distantly related to the training samples is very limited.

The purpose of this study was to shed light on some of the factors that affect the predictive performance of G-BLUP and to identify avenues by which this methodology can be improved. In particular we focused on studying how imperfect LD between markers and QTL affects the extent of shrinkage in prediction R^2^, relative to the prediction R^2^ obtained with the same sample and data structure if genotypes at causal loci were known. Several authors have studied the factors affecting prediction accuracy of G-BLUP. For instance, Goddard [28] and Daetwyler et al. [29], [30] derived formulas linking prediction R^2^ to features of the trait (e.g, *h* ^2^) of the sample (e.g., size of the training data set) and of the genome (e.g., span of LD and how this affects the number of independently segregating segments). The studies of Goddard [28] and Daetwyler [30] have a much broader scope than ours. However, because of the broader scope, their results reside on stronger assumptions and important factors affecting prediction accuracy are not accounted for. One of these assumptions is that genomes can be described as a set of independently segregating segments. This abstraction is conceptually appealing; however the abstraction is difficult to validate and the quantification of the number of independently segregating segments is controversial (various methods leading to very different values of this parameter exist, e.g., [24], [28]). In the approach presented in this study this assumption is not required.

One limitation of Goddard's approach is that it does not account for the effects of recent familial relationships (the derivations are solely based on population LD). Our approach captures, via the regression of marker-derived genomic relationships on those realized at causal loci, both effects of LD between markers and QTL as well as cosegregation between markers and QTL that occurs because of recent family relationships. On the other hand, Daetwyler's approach [30] assumes that the model accounts for all the genetic variance. We have shown that this assumption, which is not present in Goddard [28], is clearly violated in analyses involving unrelated individuals and is not part of the derivations presented in our work.

In Goddard's approach [28] the factors affecting prediction accuracy are decomposed into two components: (a) one related to the accuracy of estimates of effects and (b) one that quantifies the effects of imperfect LD between markers and QTL on prediction accuracy. In our approach, all the factors affecting the accuracy of estimates of the effects of the alleles at causal loci are captured by the R^2^ under perfect LD; and we make almost no statements about this quantity, other than the ones that follow from the properties of the multivariate normal distribution. Instead, we focus on quantifying the effects of LD on R^2^ that occurs through misspecification of TRN-TST relationships. Importantly, our simulation results show that the proposed upper bound formulas account for 80–90% of the observed shrinkage in R^2^.

In summary, our approach is complementary to that of Goddard [28] and Daetwyler [30]; we focused on a much narrower problem and by virtue of that were able to arrive at closed-form formulas that account for a sizable proportion of the shrinkage in R^2^ due to imperfect LD without making strong assumptions.

### Impacts of Genomic Relationships on Inferences and on Predictions

The ability of G-BLUP to separate true signal (**g**) from noise (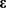) depends entirely on how well marker derived genomic relationships (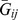) describe genetic relationships realized at unobserved causal loci (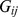). Genomic relationships at subsets of loci in the genome (e.g., markers, causal loci) can be viewed as the result of a random process with expected value given by the pedigree relationships (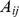) and variation due to Mendelian sampling. Because of the random nature of this process, genomic relationships vary across regions of the genome and therefore, the patterns of genomic similarity at markers and at causal loci may be different.

If the variance of the realized genomic relationships (across regions of the genome) is small relative to their expected value, the patterns of realized genomic relationships at markers will provide a good description of the patterns of realized genetic relationships at unobserved causal loci. Hill and Weir (2011) [20] have characterized various moments of the distribution of genomic relationships and concluded that the coefficient of variability decreases as the expected value, 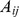, increases. Therefore, for pairs of unrelated individuals, a large coefficient of variation of genomic relationships across regions of the genome is expected. The analyses reported here support this; indeed, the regression of realized genomic relationships computed at different subsets of markers is close to one (0.98, see Table 4) for closely related individuals and very small (of the order of 0.10, see Table 4) for pairs of nominally unrelated individuals. Therefore, two contrasting situations are encountered: some of the elements of the marker derived genomic relationship matrix represent very well the true covariance function (i.e., the patterns of realized genetic relationships at observed causal loci) but others (all the off-diagonal elements corresponding to distant relatives and to pairs of unrelated individuals) show patterns of realized genomic relationships that do not describe well the patterns of realized genetic relationships at causal loci. This has direct and different impacts on estimation of variance parameters and on PA, because variance parameters and PA are driven, in part, by different forces. To illustrate with an extreme scenario, suppose that **G**, the matrix of realized genomic relationships at causal loci, is diagonal (i.e., all off-diagonal terms of **G** equal zero). In this case, it would still be possible to estimate variance parameters and genomic heritability (simply based on the fact that the diagonal elements of **G** are not constant). Yet, the prediction accuracy for phenotypes in the TST data set will be null because all the off-diagonals of **G** are equal to zero.

In this study we have chosen to center and to standardize markers using estimates of allele frequency derived from the sample. As stated, centering does not have an effect on predictions or on estimates of variance parameters [25], provided that the model contains an intercept. On the other hand, standardization can have an effect. When markers are standardized to unit variance, the relative contributions of markers to the genomic relationship matrix are the same. This is good practice if it enhances the ability of marker derived genomic relationships to describe the patterns of genetic similarity realized at causal loci. If the distribution of allele frequency at causal loci has a higher representation in the low minor allele frequency spectrum than the one observed at the markers, or if the size of effects is inversely related to minor allele frequency, then standardization may reduce the extent of missing heritability and may improve prediction accuracy.

### Estimation of Proportion of Missing Heritability Using G-BLUP Methods

The results of the simulation study indicate that when markers and QTL are in perfect LD, no missing heritability is observed, as expected. This holds regardless of whether the training sample comprises data from related or unrelated individuals. When markers and QTL are in imperfect LD two contrasting situations were encountered: (a) with family data no missing heritability was observed, and (b) with unrelated individuals, we either observed a small extent of missing heritability (when markers and QTL were sampled from the same distribution of loci, the RAND scenario) or a greater extent of missing heritability (this happened when the distribution of allele frequency at markers and causal loci was different, the Low-MAF scenario). The estimates of variance components and of genomic heritability for human height reported here are consistent with previous results for this trait. In other words, no missing heritability was observed in the analysis of family data [8] and a great extent of missing heritability (roughly 50%) was observed with unrelated individuals [7].

### Prediction Accuracy with Related and Unrelated Individuals Using G-BLUP

Predictions based on G-BLUP are weighted averages of phenotypes in the TRN data set (see, eq. 4). The weights are heavily determined by the realized TST-TRN genomic relationships (i.e., the off-diagonal entries of **G**). Therefore the PA that can be derived from G-BLUP is highly dependent on the magnitude of these coefficients and on the extent to which marker derived genomic relationships represent the underlying patterns of genetic similarity realized at causal loci. Using standard properties of the multivariate normal distribution one can derive closed-form expressions for prediction error variances and for prediction R^2^ (see Supplementary Methods). These expressions are valid **if the model holds**. This requires, among other things, that the markers used to compute genomic relationships are in perfect LD with genotypes at causal loci. Under such conditions, prediction R^2^ has an upper bound given by an index that is the product of the heritability of the trait times a weighted sum of squares of the realized genomic relationships between the individuals used for TRN and those in the TST data set (see eq. 5). The expected value of realized genomic relationships is given by the pedigree derived additive relationships. For distantly related individuals the expected value of genomic relationships is small and, consequently, data from unrelated individuals are expected to contribute little to prediction accuracy. Nevertheless, if the model holds, PA is anticipated to increase monotonically with the size of the TRN data set (each additional phenotype in the TRN data set brings additional information) and, asymptotically prediction R^2^ converges to the heritability of the trait. However, this does not occur **when markers and QTL are in imperfect LD**. Indeed, under imperfect LD, prediction R^2^ can have an upper bound that is much lower than the heritability of the trait. Assuming a linear relationship between the realized genomic relationships at markers and at causal loci, an upper bound to prediction R^2^ under imperfect LD between markers and QTL (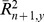) was derived (see expression 7). This upper bound is given by the product of two terms : (a) the R^2^ that can be obtained (using the same TRN sample) if markers and QTL were in perfect LD 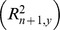 and (b) a coefficient 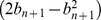 that depends on the coefficient of linear regression between TRN-TST realized genomic relationships at markers and those at causal loci 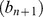. This result was derived assuming that realized genomic relationships at causal loci in the TRN data set are known, and therefore, 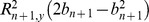 represents an upper bound on prediction 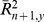 under imperfect LD.

The regression coefficient 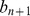 drives the size of the reduction factor on prediction R^2^. When the TRN and TST data set are related due to close familial relationships, the regression of genomic relationships at markers on those at causal loci 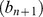 is moderately high (e.g., of the order of 0.8–0.9 for pairs of related individuals, or of the order of 0.35 when we consider a mixture of both related and unrelated individuals as in the FHS, see Table 3). Using a value of 0.35 (average 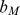 for the FHS) the minimum expected reduction factor in prediction R^2^ due to imperfect LD, 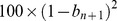, is of the order of 40–50%. On the other hand, when TRN and TST data sets are composed of nominally unrelated individuals, the regression is much smaller (of the order of 0.1). A large reduction factor in prediction R^2^ is therefore predicted (of the order of 80% computed as 100×[1–2×0.1+0.1^2^]). Importantly, the minimum shrinkage in R^2^ predicted by our formula matched very closely the observed shrinkage due to imperfect LD estimated in the simulation (roughly, the minimum shrinkage factor was 80–90% of the observed shrinkage in R^2^, see Table 3).

The maximum R^2^ that can be attained under perfect LD (assuming infinitely large samples and that the model holds) is *h* ^2^, the heritability of the trait. Imperfect LD between markers and QTL induces shrinkage in R^2^; in case of data sets of nominally unrelated individuals similar to GEN a minimum shrinkage in R^2^ of 80% is anticipated; therefore, the expected asymptotic upper bound for R^2^ is 20% of *h* ^2^, or 16% in the case of height. This estimate applies to data sets of similar characteristics that the GEN data set. Prediction problems involving individuals that are less (more) distantly related than the average individual in GEN are expected to have a lower (higher) upper bound on R^2^. Similarly, our estimates reflect the specifics of the SNP chip used and how genomic relationships were computed.

In finite samples, as pointed out in previous studies [28]–[31], estimation errors in marker effects will reduce the perfect LD R^2^ to values smaller than *h* ^2^. Some proposed formulas for the expected value of R^2^ under perfect LD take the forms 
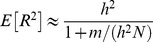
 [31], or 
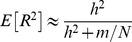
 [30], where *m* is the number of independent causal loci and *N* is the number of records in the training data set. These formulas could be used to obtain a reference for the expected R^2^ under perfect LD. However, the derivation of these formulas assumes that genotypes at causal loci are fully orthogonal. We applied these formulas using *m = 5,000*, 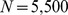 and 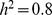, the setting of our simulation if we assume that causal loci are in linkage equilibrium, and obtained R^2^ values of 0.47 and 0.37 using the formulas suggested in [30] and [31], respectively. These values are lower than those obtained in our simulation for GEN, where R^2^ under perfect LD ranged between 0.52–0.54.

GENEVA and the FHS contain samples drawn from relatively homogeneous populations. On the other hand, when allele frequencies vary across subpopulations, so does the relative contribution of each locus affecting the trait to genetic variance in each of the subpopulations. This raises the question of what estimates of allele frequencies should one use when analyzing data involving different subpopulations. In the present study this was not an issue because the correlation of estimates of allele frequencies derived from GEN and FHM was virtually 1 (0.99). However, when this is not the case, if genomic relationships are scaled with estimates of allele frequencies derived from the entire sample, then marker derived genomic relationships will provide a poorer description of the realized genetic relationships in each of the sub-populations. This may result in a lower estimate of 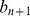 and a much higher R^2^-shrinkage factor.

Both FHS and GEN, especially the former, show some degree of population stratification, as judged by the inspection of the loadings of the 1^st^ two eigenvectors derived from **G**. However the cumulative proportion of variance explained by the first two eigenvectors was relatively small. In the presence of stratification, there may be reasons to remove between cluster variability, and to obtain within cluster estimates of variance components and of prediction accuracy. Following the approach used by Janss and coauthors [32] one could derive genomic relationships that do not include the contribution to genetic similarity of the 1^st^ k principal components of **G**. The use of such genomic relationships would yield a within cluster estimate of 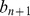. These estimates can be plugged into the equations presented here to derive an upper bound on prediction R^2^ that does not account for genetic similarity attributable to substructure.

### Implications for Data Analysis

The effectiveness of G-BLUP depends critically on the extent to which marker derived genomic relationships reflect the patterns of realized genetic relationships at causal loci. The size of the coefficient of variation of realized genomic relationships across regions of the genome depends on the number of independently segregating segments among the pair of individuals whose realized genomic relationship we wish to assess. For pairs of unrelated individuals this is largely controlled by the span of LD in the population. For pairs of related individuals this is largely controlled by within family disequilibrium. In animal and plant breeding populations G-BLUP has exhibited very good predictive performance because the two conditions needed for G-BLUP to perform well are generally met: LD span over long regions and data include highly related individuals. Under these conditions variable selection is difficult to perform and may not be needed because the patterns of genetic similarity realized at markers and at causal loci are similar.

However, the analysis of human data from unrelated individuals represents the exact opposite situation. Here LD spans over shorter regions [33] and within family disequilibrium cannot be exploited. Under these conditions the use of markers that are in imperfect LD with QTL results in very low prediction accuracy of G-BLUP. Variable selection constitutes a natural way of increasing the extent of LD between markers and QTL. However, for complex traits, stringent variable selection can induce poor coverage of regions with small, but not negligible, contribution to variance. Therefore, we are faced with the need for finding an appropriate balance: as variable selection becomes more stringent, LD between markers and QTL increases, but the some proportion of the variance contributed by QTL of small effects may be lost. The appropriate balance will likely depend on the genetic architecture of the trait but also, importantly, on features of the sample. With family data, the benefits of variable selection are relatively small. However with unrelated individuals, variable selection, including large numbers of markers (e.g., 5 K top SNPs), or perhaps better some form of smooth differential weighting of the contribution of individual markers to genomic relationships, seems to be an effective way of improving prediction accuracy. This could be done either combining information from a prior study, as implemented in this article, or using methods that perform variable selection and differential reduction of estimates of effects simultaneously. The literature on WGR offers several penalized and Bayesian methods that can achieve this goal. The application of these methods to plant and animal breeding data has not shown marked improved gains in PA relative to G-BLUP. However, for the reasons discussed in this paper, we anticipate that the situation may be different when these methods are applied to the analysis and prediction of complex traits using data from unrelated individuals.

In conclusion, we have provided an analytical framework to quantify the maximum prediction R^2^ that can be attained using G-BLUP and have compared the properties of G-BLUP in samples of related and unrelated individuals. The analytical expressions derived are consistent with our simulation and empirical results and suggest that the analysis of nominally unrelated individuals presents a number of challenges that standard G-BLUP does not address. These can be partly met by incorporating prior knowledge of the relative importance of SNPs for a given trait. Further research will be required to optimize the modeling of such prior knowledge towards improved trait prediction.

## Supporting Information

Figure S1 Squared-correlation between genotypes at adjacent markers observed in the FHS (vertical axis) and GEN datasets. The average inter-marker distance in the platform was 7.2 kb The blue lines gives the median squared-correlation in both datasets.
(PDF)

Figure S2 Average squared correlation between genotypes at various lags (number of markers in between the two used to compute the squared correlation) observed in FHS (dots) and GEN (line). The average inter-marker distance in the platform was 7.2 kb.
(PDF)

Table S1 Heritability estimates by dataset, simulation scenario and genetic information used.
(PDF)

Table S2 R-squared (R^2^) between realized and predicted phenotype in training datasets, by dataset, simulation scenario and genetic information used for analysis.
(PDF)

Table S3 R-Squared (R^2^) between realized and predicted phenotype in testing datasets, by dataset, simulation scenario and genetic information used for analysis.
(PDF)

Table S4 R-squared (R^2^) between realized and predicted genetic value in training datasets, by dataset, simulation scenario and genetic information used for analysis.
(PDF)

Table S5 R-Squared (R^2^) between realized and predicted genetic value in testing datasets, by dataset, simulation scenario and genetic information used for analysis.
(PDF)

Text S1 Includes the analytical derivations leading to the R-squared formulas presented in the article, and details of the methods used to estimate variance components and for prediction.
(PDF)
