# Supplementary material for: Prediction of Complex Human Traits Using the Genomic Best Linear Unbiased Predictor
Source: PLoS Genet. 2013 Jul 11;9(7):e1003608. doi: 10.1371/journal.pgen.1003608 (PMC3708840; doi:10.1371/journal.pgen.1003608)
Supplement: Figure S1 — Squared-correlation between genotypes at adjacent markers observed in the FHS (vertical axis) and GEN datasets. The average inter-marker distance in the platform was 7.2 kb The blue lines gives the median squared-correlation in both datasets. (PDF) [file pgen.1003608.s001.pdf]

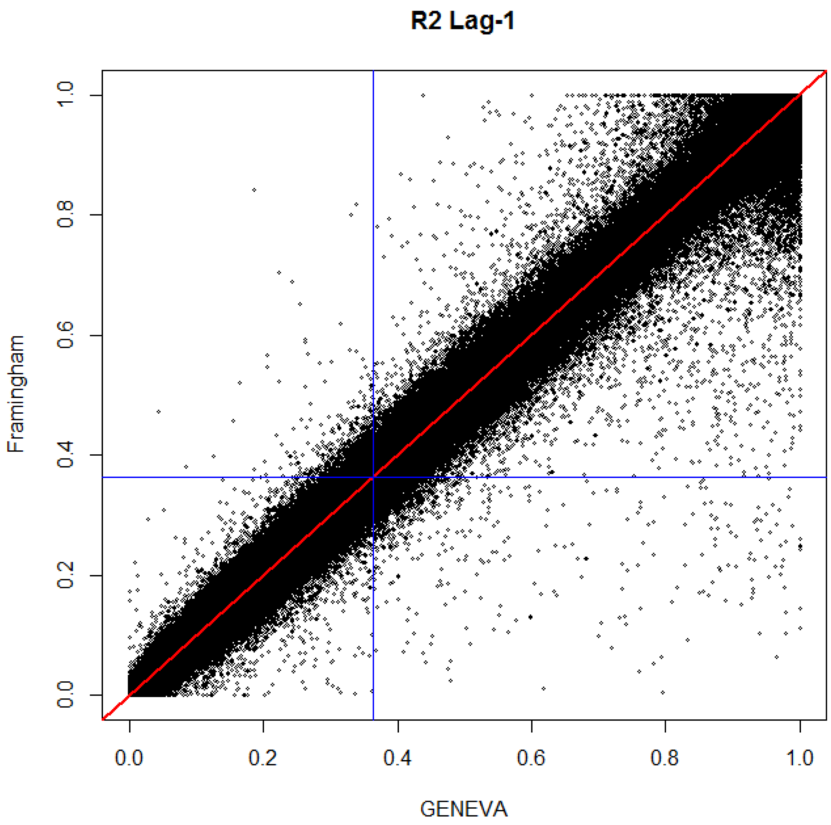

Figure S1. Squared-correlation between genotypes at adjacent markers observed in the FHS (vertical axis) and GEN datasets. The average inter-marker distance in the platform was 7.2kb The blue lines gives the median squared-correlation in both datasets.
