# Supplementary material for: Prediction of Complex Human Traits Using the Genomic Best Linear Unbiased Predictor
Source: PLoS Genet. 2013 Jul 11;9(7):e1003608. doi: 10.1371/journal.pgen.1003608 (PMC3708840; doi:10.1371/journal.pgen.1003608)
Supplement: Figure S2 — Average squared correlation between genotypes at various lags (number of markers in between the two used to compute the squared correlation) observed in FHS (dots) and GEN (line). The average inter-marker distance in the platform was 7.2 kb. (PDF) [file pgen.1003608.s002.pdf]

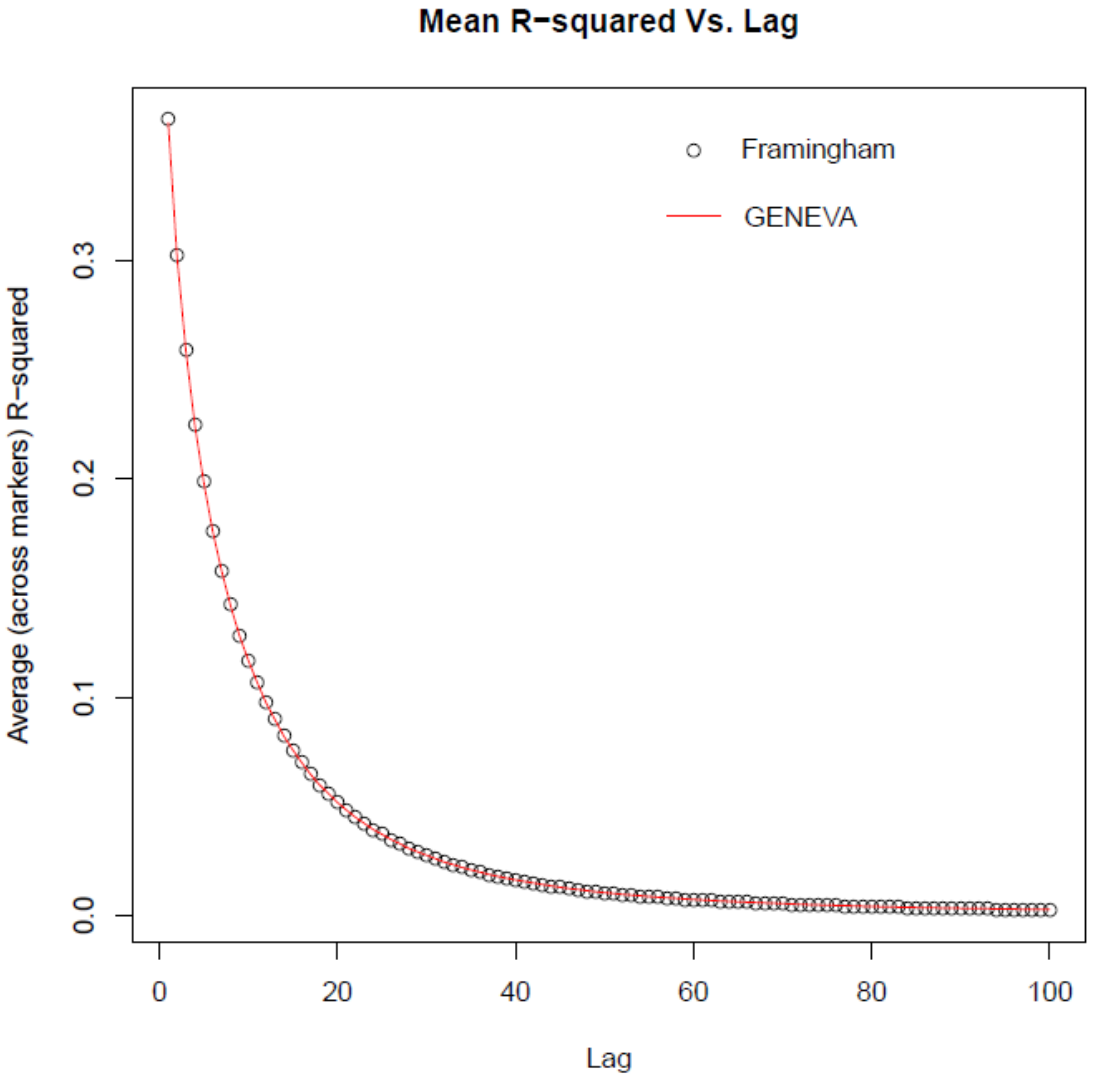

Figure S2. Average squared correlation between genotypes at various lags (number of markers in between the two used to compute the squared correlation) observed in FHS (dots) and GEN (line). The average inter-marker distance in the platform was 7.2 kb.
