# Supplementary material for: Prediction of Complex Human Traits Using the Genomic Best Linear Unbiased Predictor
Source: PLoS Genet. 2013 Jul 11;9(7):e1003608. doi: 10.1371/journal.pgen.1003608 (PMC3708840; doi:10.1371/journal.pgen.1003608)
Supplement: Table S1 — Heritability estimates by dataset, simulation scenario and genetic information used. (PDF) [file pgen.1003608.s003.pdf]

Table S1. Estimates of genomic heritability, h_G^2 , by data set, simulation scenario, genetic information used and Monte Carlo replicate.

Dataset	FRAMINGHAM						GENEVA			
Scenario	RAND			LOW-MAF			RAND		LOW-MAF	
Information	QTL	Markers	Pedigree	QTL	Markers	Pedigree	QTL	Markers	QTL	Markers
Rep-1	.757	.781	.767	.766	.762	.769	.755	.706	.768	.552
Rep-2	.775	.751	.742	.779	.713	.714	.774	.668	.777	.562
Rep-3	.781	.790	.796	.785	.788	.798	.778	.800	.783	.620
Rep-4	.780	.779	.774	.770	.736	.765	.770	.751	.784	.636
Rep-5	.779	.761	.751	.780	.741	.737	.775	.724	.784	.703
Rep-6	.766	.764	.750	.772	.776	.787	.756	.727	.757	.656
Rep-7	.776	.806	.805	.789	.761	.775	.776	.677	.775	.585
Rep-8	.785	.772	.764	.778	.747	.761	.772	.679	.777	.582
Rep-9	.769	.786	.769	.774	.760	.769	.755	.780	.774	.552
Rep-10	.768	.735	.725	.782	.744	.729	.752	.748	.775	.595
Rep-11	.775	.789	.770	.765	.723	.729	.765	.733	.769	.461
Rep-12	.767	.760	.726	.772	.755	.760	.785	.775	.777	.602
Rep-13	.779	.784	.760	.792	.773	.800	.795	.733	.770	.532
Rep-14	.805	.800	.782	.793	.723	.718	.787	.721	.778	.571
Rep-15	.781	.749	.761	.779	.759	.766	.767	.774	.779	.551
Rep-16	.774	.786	.774	.768	.753	.755	.784	.750	.786	.533
Rep-17	.778	.757	.737	.781	.710	.724	.778	.713	.768	.575
Rep-18	.788	.778	.759	.773	.754	.776	.778	.795	.772	.458
Rep-19	.776	.796	.787	.769	.735	.747	.767	.675	.769	.567
Rep-20	.776	.776	.772	.787	.754	.768	.766	.722	.784	.617
Rep-21	.771	.775	.772	.779	.750	.736	.772	.788	.756	.504
Rep-22	.757	.753	.762	.780	.734	.726	.779	.787	.769	.496
Rep-23	.771	.767	.754	.775	.757	.760	.772	.687	.797	.517
Rep-24	.772	.781	.762	.777	.742	.769	.769	.726	.777	.544
Rep-25	.777	.795	.784	.782	.768	.783	.786	.806	.779	.540
Rep-26	.762	.740	.721	.771	.747	.743	.772	.693	.765	.646
Rep-27	.781	.784	.766	.782	.758	.770	.768	.749	.775	.590
Rep-28	.775	.789	.791	.777	.736	.729	.763	.709	.782	.652
Rep-29	.772	.768	.759	.777	.741	.751	.780	.752	.777	.649
Rep-30	.769	.768	.770	.773	.738	.740	.779	.772	.778	.554
**Average**	.775	.774	.764	.777	.748	.755	.773	.737	.775	.573
**SD**	.009	.018	.020	.007	.018	.023	.010	.040	.008	.058
