# Supplementary material for: Prediction of Complex Human Traits Using the Genomic Best Linear Unbiased Predictor
Source: PLoS Genet. 2013 Jul 11;9(7):e1003608. doi: 10.1371/journal.pgen.1003608 (PMC3708840; doi:10.1371/journal.pgen.1003608)
Supplement: Table S2 — R-squared (R2) between realized and predicted phenotype in training datasets, by dataset, simulation scenario and genetic information used for analysis. (PDF) [file pgen.1003608.s004.pdf]

Table S2. R-squared (R^2) between realized and predicted phenotype in training data sets, by data set, simulation scenario, genetic information used and Monte Carlo replicate.

Dataset	FRAMINGHAM						GENEVA			
Scenario	RAND			LOW-MAF			RAND		LOW-MAF	
Information	QTL	Markers	Pedigree	QTL	Markers	Pedigree	QTL	Markers	QTL	Markers
Rep-1	.905	.964	.965	.908	.958	.966	.922	.989	.925	.976
Rep-2	.914	.955	.958	.914	.942	.950	.931	.986	.928	.973
Rep-3	.916	.967	.973	.920	.966	.974	.933	.995	.933	.982
Rep-4	.919	.964	.968	.910	.950	.966	.929	.992	.932	.985
Rep-5	.917	.959	.962	.916	.952	.957	.933	.990	.933	.989
Rep-6	.909	.959	.961	.913	.963	.971	.923	.990	.919	.985
Rep-7	.918	.971	.975	.921	.958	.968	.931	.988	.929	.979
Rep-8	.919	.962	.965	.914	.953	.964	.930	.987	.929	.977
Rep-9	.914	.965	.966	.912	.958	.966	.922	.994	.927	.974
Rep-10	.910	.949	.953	.916	.952	.954	.921	.992	.928	.981
Rep-11	.915	.967	.966	.907	.945	.955	.927	.991	.925	.963
Rep-12	.908	.958	.954	.911	.956	.963	.937	.994	.930	.978
Rep-13	.917	.966	.964	.923	.962	.974	.941	.991	.926	.970
Rep-14	.932	.970	.970	.921	.945	.951	.937	.990	.930	.978
Rep-15	.918	.954	.964	.915	.957	.965	.929	.994	.929	.974
Rep-16	.916	.965	.967	.909	.955	.962	.936	.992	.933	.974
Rep-17	.916	.956	.956	.915	.940	.952	.934	.990	.924	.980
Rep-18	.923	.963	.963	.913	.955	.968	.933	.995	.926	.963
Rep-19	.915	.969	.971	.908	.949	.960	.928	.986	.925	.975
Rep-20	.917	.962	.967	.919	.956	.966	.928	.990	.933	.980
Rep-21	.912	.962	.967	.915	.955	.957	.931	.994	.920	.963
Rep-22	.906	.955	.964	.914	.950	.954	.935	.993	.924	.966
Rep-23	.912	.960	.961	.912	.957	.963	.931	.987	.939	.970
Rep-24	.910	.964	.965	.914	.952	.966	.929	.990	.930	.959
Rep-25	.915	.968	.970	.917	.960	.970	.936	.995	.931	.973
Rep-26	.907	.951	.952	.911	.954	.958	.930	.989	.924	.986
Rep-27	.919	.965	.965	.918	.957	.966	.929	.992	.928	.980
Rep-28	.917	.966	.972	.914	.950	.954	.927	.988	.932	.984
Rep-29	.913	.960	.963	.914	.951	.961	.935	.992	.931	.981
Rep-30	.911	.960	.966	.911	.950	.957	.934	.993	.930	.976
**Average**	.915	.962	.964	.914	.954	.962	.931	.991	.928	.976
**SE**	.005	.006	.006	.004	.006	.007	.005	.003	.004	.008
