# Supplementary material for: Prediction of Complex Human Traits Using the Genomic Best Linear Unbiased Predictor
Source: PLoS Genet. 2013 Jul 11;9(7):e1003608. doi: 10.1371/journal.pgen.1003608 (PMC3708840; doi:10.1371/journal.pgen.1003608)
Supplement: Table S3 — R-Squared (R2) between realized and predicted phenotype in testing datasets, by dataset, simulation scenario and genetic information used for analysis. (PDF) [file pgen.1003608.s005.pdf]

Table S3. R-Squared (R^2) between realized and predicted phenotype in testing data sets, by data set, simulation scenario, genetic information used and Monte Carlo replicate.

Dataset	FRAMINGHAM						GENEVA			
Scenario	RAND			LOW-MAF			RAND		LOW-MAF	
Information	QTL	Markers	Pedigree	QTL	Markers	Pedigree	QTL	Markers	QTL	Markers
Rep-1	.600	.373	.305	.490	.191	.170	.546	.069	.529	.038
Rep-2	.524	.270	.236	.561	.226	.191	.538	.029	.535	.039
Rep-3	.463	.214	.148	.556	.222	.195	.558	.067	.541	.039
Rep-4	.501	.234	.207	.542	.254	.252	.539	.073	.522	.034
Rep-5	.571	.239	.235	.620	.254	.238	.494	.069	.508	.058
Rep-6	.586	.316	.258	.544	.245	.217	.517	.097	.503	.070
Rep-7	.531	.333	.259	.554	.263	.216	.501	.084	.583	.040
Rep-8	.586	.332	.305	.531	.216	.191	.528	.042	.542	.036
Rep-9	.523	.208	.177	.584	.237	.250	.545	.066	.548	.050
Rep-10	.486	.165	.103	.528	.241	.242	.475	.031	.552	.050
Rep-11	.522	.255	.240	.556	.233	.203	.526	.124	.514	.036
Rep-12	.525	.278	.246	.560	.236	.213	.502	.105	.547	.065
Rep-13	.538	.233	.233	.550	.205	.194	.495	.073	.533	.044
Rep-14	.592	.295	.207	.536	.176	.133	.513	.074	.521	.036
Rep-15	.542	.254	.228	.557	.226	.212	.509	.063	.518	.019
Rep-16	.571	.340	.291	.537	.218	.214	.556	.069	.585	.042
Rep-17	.570	.257	.244	.543	.245	.234	.464	.025	.554	.060
Rep-18	.495	.264	.232	.535	.243	.221	.521	.050	.531	.044
Rep-19	.493	.271	.207	.565	.212	.198	.473	.095	.507	.023
Rep-20	.629	.344	.299	.538	.280	.262	.460	.058	.522	.059
Rep-21	.540	.216	.169	.530	.307	.289	.499	.047	.473	.018
Rep-22	.516	.192	.146	.527	.243	.249	.566	.075	.563	.084
Rep-23	.565	.293	.258	.545	.248	.234	.569	.077	.579	.037
Rep-24	.532	.232	.206	.506	.212	.213	.535	.101	.500	.091
Rep-25	.491	.234	.169	.578	.232	.221	.542	.053	.527	.077
Rep-26	.558	.247	.200	.575	.306	.264	.468	.086	.540	.032
Rep-27	.587	.263	.235	.573	.265	.261	.548	.087	.536	.045
Rep-28	.595	.280	.229	.600	.249	.226	.502	.069	.544	.062
Rep-29	.548	.234	.203	.544	.280	.288	.531	.079	.548	.091
Rep-30	.561	.238	.218	.562	.243	.221	.494	.081	.571	.049
**Average**	.545	.263	.223	.551	.240	.224	.517	.071	.536	.049
**SE**	.040	.048	.047	.026	.029	.033	.031	.023	.026	.019
