# Supplementary material for: Prediction of Complex Human Traits Using the Genomic Best Linear Unbiased Predictor
Source: PLoS Genet. 2013 Jul 11;9(7):e1003608. doi: 10.1371/journal.pgen.1003608 (PMC3708840; doi:10.1371/journal.pgen.1003608)
Supplement: Table S4 — R-squared (R2) between realized and predicted genetic value in training datasets, by dataset, simulation scenario and genetic information used for analysis. (PDF) [file pgen.1003608.s006.pdf]

Table S4. R-squared (R^2) between realized and predicted genetic value in training data sets, by data set, simulation scenario, genetic information used and Monte Carlo replicate.

Dataset	FRAMINGHAM						GENEVA			
Scenario	RAND			LOW-MAF			RAND		LOW-MAF	
Information	QTL	Markers	Pedigree	QTL	Markers	Pedigree	QTL	Markers	QTL	Markers
Rep-1	.871	.827	.824	.870	.820	.818	.848	.793	.856	.791
Rep-2	.868	.824	.817	.869	.818	.814	.848	.794	.855	.788
Rep-3	.861	.817	.813	.879	.833	.830	.846	.798	.859	.802
Rep-4	.866	.823	.819	.870	.818	.820	.856	.804	.856	.799
Rep-5	.873	.827	.822	.876	.830	.827	.862	.811	.858	.807
Rep-6	.856	.810	.805	.869	.823	.820	.843	.787	.842	.782
Rep-7	.878	.834	.830	.876	.827	.827	.854	.800	.860	.797
Rep-8	.865	.823	.818	.873	.825	.824	.855	.800	.864	.804
Rep-9	.876	.831	.827	.864	.815	.813	.845	.793	.848	.783
Rep-10	.865	.822	.816	.869	.822	.818	.850	.794	.853	.792
Rep-11	.869	.828	.823	.861	.810	.809	.852	.799	.851	.781
Rep-12	.862	.818	.812	.869	.823	.822	.853	.802	.859	.798
Rep-13	.862	.819	.814	.868	.824	.823	.861	.812	.858	.792
Rep-14	.879	.841	.837	.870	.820	.819	.862	.813	.874	.808
Rep-15	.864	.822	.819	.869	.822	.820	.858	.807	.854	.790
Rep-16	.872	.831	.827	.867	.820	.817	.861	.812	.863	.801
Rep-17	.871	.829	.822	.866	.814	.812	.858	.810	.857	.796
Rep-18	.879	.837	.832	.874	.829	.828	.854	.806	.854	.786
Rep-19	.874	.828	.823	.865	.814	.813	.841	.790	.851	.788
Rep-20	.873	.832	.828	.869	.823	.823	.855	.803	.863	.806
Rep-21	.854	.811	.807	.869	.820	.816	.858	.804	.847	.774
Rep-22	.859	.817	.812	.867	.818	.814	.856	.808	.852	.783
Rep-23	.872	.829	.825	.871	.825	.823	.858	.805	.863	.800
Rep-24	.857	.812	.806	.864	.813	.813	.846	.794	.855	.786
Rep-25	.865	.820	.817	.867	.817	.818	.856	.807	.863	.794
Rep-26	.866	.822	.817	.873	.825	.822	.861	.808	.859	.801
Rep-27	.867	.827	.822	.874	.825	.824	.861	.810	.859	.801
Rep-28	.871	.828	.823	.871	.820	.818	.850	.801	.863	.803
Rep-29	.872	.828	.823	.868	.823	.821	.854	.805	.869	.809
Rep-30	.862	.820	.814	.860	.807	.806	.854	.806	.855	.792
**Average**	.868	.825	.820	.869	.821	.819	.854	.803	.857	.794
**SE**	.007	.007	.008	.004	.006	.006	.006	.007	.007	.009
