# Supplementary material for: Prediction of Complex Human Traits Using the Genomic Best Linear Unbiased Predictor
Source: PLoS Genet. 2013 Jul 11;9(7):e1003608. doi: 10.1371/journal.pgen.1003608 (PMC3708840; doi:10.1371/journal.pgen.1003608)
Supplement: Table S5 — R-Squared (R2) between realized and predicted genetic value in testing datasets, by dataset, simulation scenario and genetic information used for analysis. (PDF) [file pgen.1003608.s007.pdf]

Table S5. R-Squared (R^2) between realized and predicted genetic value in testing data sets, by data set, simulation scenario, genetic information used and Monte Carlo replicate.

Dataset	FRAMINGHAM						GENEVA			
Scenario	RAND			LOW-MAF			RAND		LOW-MAF	
Information	QTL	Markers	Pedigree	QTL	Markers	Pedigree	QTL	Markers	QTL	Markers
Rep-1	.703	.438	.363	.641	.236	.201	.675	.084	.665	.033
Rep-2	.665	.332	.307	.694	.265	.244	.617	.040	.681	.053
Rep-3	.597	.258	.179	.698	.273	.238	.671	.068	.653	.042
Rep-4	.652	.314	.273	.692	.354	.333	.672	.114	.651	.056
Rep-5	.697	.291	.284	.741	.299	.270	.640	.076	.657	.073
Rep-6	.701	.381	.313	.694	.287	.258	.665	.136	.640	.091
Rep-7	.671	.394	.310	.686	.312	.264	.645	.085	.703	.042
Rep-8	.721	.428	.378	.713	.303	.245	.644	.051	.680	.038
Rep-9	.653	.272	.225	.712	.294	.288	.641	.079	.670	.063
Rep-10	.634	.228	.142	.674	.317	.315	.611	.035	.680	.037
Rep-11	.688	.335	.312	.687	.266	.226	.639	.142	.641	.045
Rep-12	.675	.372	.324	.691	.306	.288	.626	.128	.703	.061
Rep-13	.695	.304	.300	.663	.254	.242	.654	.113	.674	.059
Rep-14	.693	.330	.239	.666	.241	.185	.615	.069	.688	.036
Rep-15	.664	.308	.287	.717	.298	.278	.608	.076	.637	.021
Rep-16	.691	.434	.378	.677	.259	.248	.685	.092	.721	.059
Rep-17	.703	.320	.287	.677	.319	.304	.617	.043	.691	.067
Rep-18	.650	.310	.256	.669	.286	.271	.624	.056	.680	.065
Rep-19	.605	.318	.227	.673	.264	.240	.613	.125	.628	.028
Rep-20	.725	.394	.351	.691	.322	.300	.613	.079	.670	.061
Rep-21	.668	.256	.199	.661	.366	.355	.633	.068	.603	.034
Rep-22	.661	.270	.200	.665	.312	.301	.669	.078	.661	.105
Rep-23	.729	.400	.356	.678	.336	.324	.667	.095	.704	.051
Rep-24	.713	.326	.276	.660	.265	.264	.683	.110	.640	.111
Rep-25	.632	.278	.216	.699	.287	.273	.689	.062	.661	.083
Rep-26	.687	.304	.235	.685	.374	.335	.604	.105	.688	.041
Rep-27	.704	.353	.318	.711	.323	.314	.680	.105	.706	.055
Rep-28	.723	.337	.270	.707	.295	.252	.629	.079	.674	.097
Rep-29	.676	.279	.241	.682	.338	.353	.661	.080	.675	.107
Rep-30	.699	.275	.248	.725	.308	.286	.621	.076	.677	.054
**Average**	.679	.328	.277	.688	.299	.276	.644	.085	.670	.059
**SE**	.034	.056	.060	.022	.035	.042	.027	.028	.026	.024
