# Supplementary material for: Prediction of Complex Human Traits Using the Genomic Best Linear Unbiased Predictor
Source: PLoS Genet. 2013 Jul 11;9(7):e1003608. doi: 10.1371/journal.pgen.1003608 (PMC3708840; doi:10.1371/journal.pgen.1003608)
Supplement: Text S1 — Includes the analytical derivations leading to the R-squared formulas presented in the article, and details of the methods used to estimate variance components and for prediction. (PDF) [file pgen.1003608.s008.pdf]

Supplementary Methods

Sections 1 and 2 give a detailed account of the model and of the analytical derivations presented in the main manuscript. All the results presented in Section 1 and 2.1 are well established in the literature; we have tried to provide detailed derivations based on properties of the multivariate normal density. Readers interested on further details are referred to [1,2]. To the best of our knowledge, the results presented on section 2.2., prediction R-squared under imperfect LD between markers and genotypes at causal loci, are novel. Finally **Section 3** gives a complete account of the Bayesian implementation used to fit models.

1. Genomic BLUP (Best Linear Unbiased Predictor)

Consider a linear regression on marker covariates of the form,

$$y_i = \sum_{l=1}^{p} w_{il}\beta_l + \varepsilon_i \,, \qquad \text{(S.1)}$$

where, y_i represents a phenotypic measure taken on the ith individual in the sample ($i=1,\ldots,n$), w_{il} are marker covariates ($l=1,\ldots,p$ markers), β_l are marker effects and e_i are model residuals. Here, we assume that phenotypes are centered, $\sum_{i=1}^{n} y_i = 0$, and that marker covariates are centered and standardized to unit variance, $w_{il} = \frac{(x_{il} - 2\theta_l)}{\sqrt{2\theta_l(1-\theta_l)}}$, where $x_{il} \in (0,1,2)$ counts the number of copies of one of the alleles observed at the lth locus of the ith individual and θ_l is an estimate of the frequency of the allele coded as one at the lth locus. Centering and standardization are not strictly needed for the arguments we outline to hold, but the presentation is greatly facilitated. Stacking all the equations for individuals 1 to n, we have:

$$\mathbf{y} = \mathbf{W}\boldsymbol{\beta} + \boldsymbol{\varepsilon} \qquad \text{(S.2)}$$

where $\mathbf{y} = \{y_i\}$, $\boldsymbol{\beta} = \{\beta_l\}$ and $\boldsymbol{\varepsilon} = \{\varepsilon_i\}$ represent vectors of phenotypes, marker effects and model residuals and $\mathbf{W} = \{w_{il}\}$ is a matrix of marker covariates. In a standard Bayesian Gaussian Regression marker effects are assumed to be IID (independent and identically distributed) draws from a normal

density $\beta_l \overset{IID}{\sim} N\left(0,\sigma_\beta^2\right)$, and model residuals are also assumed to be IID normal, $\varepsilon_i \overset{IID}{\sim} N\left(0,\sigma_\varepsilon^2\right)$, independent of marker effects. Here, σ_β^2 and σ_ε^2 represent the prior variance of marker effects and the variance of model residuals.

Model. The linear score $u_i = \sum_{l=1}^{p} w_{il}\beta_l$, the 'genomic value', represents the expected value of the ith phenotype given marker genotypes and marker effects. Replacing this in the data equation (S.1) we arrive at the following random effects model $y_i = u_i + \varepsilon_i$, or in matrix notation

$$\mathbf{y} = \mathbf{u} + \boldsymbol{\varepsilon} \qquad \text{(S.3)}$$

where, $\mathbf{u} = (u_1,...,u_n)'$. Following standard properties of the multivariate normal density it can be shown that the joint density of $\mathbf{u}$ is multivariate normal, with mean equal to zero and variance-covariance matrix proportional to $\mathbf{G} = p^{-1}\mathbf{W}\mathbf{W}'$; therefore, $\mathbf{u} \sim N\left(\mathbf{0},\mathbf{G}\sigma_u^2\right)$, with $\sigma_u^2 = p\sigma_\beta^2$.

Collecting assumptions, the joint distribution of phenotypes, genetic values and model residuals is given by:

$$\begin{bmatrix} \mathbf{u} \\ \boldsymbol{\varepsilon} \\ \mathbf{y} \end{bmatrix} \sim MVN\left[\mathbf{0}, \begin{pmatrix} \mathbf{G}\sigma_u^2 & \mathbf{0} & \mathbf{G}\sigma_u^2 \\ \mathbf{0} & \mathbf{I}\sigma_\varepsilon^2 & \mathbf{I}\sigma_\varepsilon^2 \\ \mathbf{G}\sigma_u^2 & \mathbf{I}\sigma_\varepsilon^2 & \mathbf{G}\sigma_u^2 + \mathbf{I}\sigma_\varepsilon^2 \end{pmatrix}\right] \qquad \text{(S.4)}$$

Best Linear Unbiased Predictor of Genomic Values. The conditional expectation (CE) is the best predictor in the mean-squared error sense, and in the model defined by (S.4) the CE of **u** given **y** is:

$$\begin{aligned} E[\mathbf{u}|\mathbf{y}] &= Cov[\mathbf{u},\mathbf{y}']Var[\mathbf{y}]^{-1}\mathbf{y} \\ &= \sigma_u^2\mathbf{G}\left[\mathbf{G}\sigma_u^2 + \mathbf{I}\sigma_\varepsilon^2\right]^{-1}\mathbf{y} \\ &= \mathbf{G}\left[\mathbf{G} + \mathbf{I}\lambda\right]^{-1}\mathbf{y} \\ &= \mathbf{G}\mathbf{T}\mathbf{y} \\ &= \mathbf{G}\tilde{\mathbf{y}} \qquad \text{(S.5a)} \end{aligned}$$

where $\lambda = \sigma_\varepsilon^2\sigma_u^{-2}$, $\mathbf{T} = \left[\mathbf{G} + \mathbf{I}\lambda\right]^{-1}$ is a matrix proportional to the inverse of the phenotypic variance-covariance matrix of phenotypes and $\tilde{\mathbf{y}} = \mathbf{T}\mathbf{y}$ is a vector of 'smoothed' phenotypes obtained by pre-multiplying **y** with **T**. The expected value (over possible realizations of $\mathbf{u}$ and $\boldsymbol{\varepsilon}$) of the CE formula of

(S.5a) equal's the prior expectation of $\mathbf{u}, E\{E[\mathbf{u}|\mathbf{y}]\} = E[\mathbf{GTy}] = \mathbf{GT}E[\mathbf{y}] = \mathbf{0}$; therefore predictions derived using (S.5a) are unbiased with respect to the prior expectation. Finally, the predictor in (S.5a) is linear on **y**; therefore (S.5a) is the BLUP of **u**.

The equation corresponding to the ith entry in the CE vector of (S.5) is given by

$$\begin{aligned} E[u_i|\mathbf{y}] &= \sum_{j=1}^{n} G_{ij} \sum_{k=1}^{n} T_{jk} y_k \\ &= \sum_{j=1}^{n} G_{ij} \tilde{y}_j \end{aligned} \qquad \text{(S.5b)}$$

where $\tilde{y}_j = \sum_{k=1}^{n} T_{jk} y_k$.

Best Linear unbiased Prediction of Marker Effects. In the model above, marker effects and phenotypes follow the following MVN density:

$$\begin{bmatrix} \boldsymbol{\beta} \\ \mathbf{y} \end{bmatrix} \sim MVN\left[\mathbf{0}, \begin{pmatrix} \mathbf{I}\sigma_\beta^2 & \mathbf{W}'\sigma_\beta^2 \\ \mathbf{W}\sigma_\beta^2 & \mathbf{WW}'\sigma_\beta^2 + \mathbf{I}\sigma_\varepsilon^2 \end{pmatrix}\right]$$

Therefore, the expected value of marker effects given phenotypes is:

$$\begin{aligned} E[\boldsymbol{\beta}|\mathbf{y}] &= Cov[\boldsymbol{\beta}, \mathbf{y}'] Var[\mathbf{y}]^{-1}\mathbf{y} \\ &= \sigma_\beta^2 \mathbf{W}'[\mathbf{WW}'\sigma_\beta^2 + \mathbf{I}\sigma_\varepsilon^2]^{-1}\mathbf{y} \\ &= \frac{1}{p}\sigma_u^2 \mathbf{W}'[\mathbf{G}\sigma_u^2 + \mathbf{I}\sigma_\varepsilon^2]^{-1}\mathbf{y} \\ &= \frac{1}{p}\mathbf{W}'[\mathbf{G} + \mathbf{I}\lambda]^{-1}\mathbf{y} \\ &= \frac{1}{p}\mathbf{W}'\mathbf{Ty} = \frac{1}{p}\mathbf{W}'\tilde{\mathbf{y}} \end{aligned} \qquad \text{(S.6)}$$

Above, $\sigma_\beta^2 = \sigma_u^2 / p$, $\mathbf{G} = p^{-1}\mathbf{WW}'$ and $\mathbf{T} = [\mathbf{G} + \mathbf{I}\lambda]^{-1}$. It can be shown that predictions of marker effects given by (S.6) are equivalent to the Ridge Regression [3] estimates, $\hat{\boldsymbol{\beta}} = [\mathbf{W}'\mathbf{W} + \mathbf{I}p\lambda]^{-1}\mathbf{W}'\mathbf{y}$.

Note. In the derivation of the BLUPs in (S.5) and (S.6) we have assumed that variance components are known. When variance components are unknown these can be estimated from data using various methods (e.g., Maximum Likelihood, Restricted Maximum Likelihood or Bayesian Methods, see Section 2 of this document). However, when variance components are estimated from the data, the CE

function does not have a closed form and predictions are not linear functions of the data; therefore, predictions derived when variance components are estimated from the data are not strictly BLUP anymore.

Predictions of Yet-to-be Observed Phenotypes. Consider a partition of the vectors in eq. (S.3) into two disjoint sets $\{\mathbf{y}_1, \mathbf{u}_1, \boldsymbol{\varepsilon}_1\}$ and $\{\mathbf{y}_2, \mathbf{u}_2, \boldsymbol{\varepsilon}_2\}$, pertaining to the training (TRN) and testing (TST) data sets, respectively, so that:

$$\begin{bmatrix}\mathbf{y}_1\\ \mathbf{y}_2\end{bmatrix} = \begin{bmatrix}\mathbf{u}_1\\ \mathbf{u}_2\end{bmatrix} + \begin{bmatrix}\boldsymbol{\varepsilon}_1\\ \boldsymbol{\varepsilon}_2\end{bmatrix} \qquad \text{(S.7)}$$

The joint density of phenotypes in TRN and TST data sets is then given by the following MVN density:

$$\begin{bmatrix}\mathbf{y}_1\\ \mathbf{y}_2\end{bmatrix} \sim MVN\left[\mathbf{0}, \begin{pmatrix}\mathbf{G}_{11}\sigma_u^2 + \mathbf{I}_1\sigma_\varepsilon^2 & \mathbf{G}_{12}\sigma_u^2\\ \mathbf{G}_{21}\sigma_u^2 & \mathbf{G}_{22}\sigma_u^2 + \mathbf{I}_2\sigma_\varepsilon^2\end{pmatrix}\right] \qquad \text{(S.8)}$$

where $\mathbf{G}_{11}$ and $\mathbf{G}_{22}$ represent matrices describing genomic relationships among individuals of the TRN and TST data sets, respectively, and $\mathbf{G}_{21} = \mathbf{G}'_{12}$ is a matrix describing genomic relationships between individuals in the TST and those in the TRN data set. From equation (S.8) the expected value of phenotypes in the TST data set given the phenotypes in the TRN data set is,

$$\begin{aligned} E[\mathbf{y}_2 | \mathbf{y}_1] &= Cov[\mathbf{y}_2, \mathbf{y}'_1] Var[\mathbf{y}_1]^{-1}\mathbf{y}_1 \\ &= \sigma_u^2\mathbf{G}_{21}[\sigma_u^2\mathbf{G}_{11} + \sigma_\varepsilon^2\mathbf{I}_1]^{-1}\mathbf{y}_1 \\ &= \mathbf{G}_{21}[\mathbf{G}_{11} + \mathbf{I}_1\lambda]^{-1}\mathbf{y}_1 \\ &= \mathbf{G}_{21}\mathbf{T}\mathbf{y}_1 \qquad \text{(S.9a)} \\ &= \mathbf{G}_{21}\tilde{\mathbf{y}}_1 \end{aligned}$$

Above, $\mathbf{T} = [\mathbf{G}_{11} + \mathbf{I}_1\lambda]^{-1}$ is a matrix proportional to the inverse of the (co)variance matrix of phenotypes in the TRN data set. A scalar version of (S.9a) is given by the following linear score (hereinafter, we assume that the TRN data set includes n individuals and, without loss of generality, we present prediction equations and other expression pertaining genetic values or phenotypes in the TST data set using sub-index $n+1$ to stress that such expression pertains to observations not included in the TRN data set):

$$E\left[y_{n+1}|\mathbf{y}_1\right] = \sum\nolimits_{i=1}^{n} G_{n+1,i} \sum\nolimits_{j=1}^{n} T_{ij} y_j$$
$$= \sum\nolimits_{i=1}^{n} G_{n+1,i} \tilde{y}_i \qquad \text{(S.9b)}$$

Therefore, predictions in G-BLUP are simply weighted sums of (pre-smoohted) phenotypes of the individuals in the TRN data set, with weights given by the realized genomic relationships between individuals in TRN and TST data sets $\left\{G_{n+1,i}\right\}_{i=1}^{n}$.

2. Measures of Prediction Accuracy in G-BLUP

The variance of prediction errors, or prediction error variance (PEV), is commonly used in prediction problems to assess the predictive power of an entertained model. In case of prediction of yet-to-be-observed phenotypes, variance-covariance matrices of prediction errors of genetic values and of phenotypes are given by

$$Var\left(\mathbf{u}_2 - E\left[\mathbf{u}_2|\mathbf{y}_1\right]\right) = Var\left(\mathbf{u}_2\right) + Var\left(E\left[\mathbf{u}_2|\mathbf{y}_1\right]\right) - 2Cov\left(\mathbf{u}_2, E\left[\mathbf{u}_2|\mathbf{y}_1\right]'\right)$$

and

$$Var\left(\mathbf{y}_2 - E\left[\mathbf{y}_2|\mathbf{y}_1\right]\right) = Var\left(\mathbf{y}_2\right) + Var\left(E\left[\mathbf{y}_2|\mathbf{y}_1\right]\right) - 2Cov\left(\mathbf{y}_2, E\left[\mathbf{y}_2|\mathbf{y}_1\right]'\right)$$

, respectively. The next sub-section presents expressions for prediction error variances and co-variances under the assumptions of the model.

2.1. Case 1: when genotypes at causal loci are known (i.e., perfect LD between markers and QTL)

We begin by assuming that genomic relationship matrices are computed using realized genotypes at causal loci. In this case, the model holds, and closed-form formulas for PEV and R^2 can be derived. The results that follow use (S.8) as starting point and standard properties of the multivariate normal density. A list of useful results that will be used later is given in Table S1.

Table S1. Useful Results: variances, (co)variances and prediction error variances of genetic values and of phenotypes of individuals in the testing data set.

	Genetic Values	Phenotypes
Marginal Variance	$Var(\mathbf{u}_2)=\mathbf{G}_{22}\sigma_u^2$ (S10.a)	$Var(\mathbf{y}_2)=\left[\mathbf{G}_{22}\sigma_u^2+\mathbf{I}_2\sigma_\varepsilon^2\right]$ (S10.b)
Conditional Variances	$Var(\mathbf{u}_2\vert\mathbf{y}_1)=\sigma_u^2\{\mathbf{G}_{22}-\mathbf{G}_{21}\mathbf{TG}_{12}\}$ (S10.c)	$Var(\mathbf{y}_2\vert\mathbf{y}_1)=Var(\mathbf{u}_2\vert\mathbf{y}_1)+\mathbf{I}_2\sigma_\varepsilon^2$ (S10.d)
Variance of Cond. Expectation	$Var(E[\mathbf{y}_2\vert\mathbf{y}_1])=Var(E[\mathbf{u}_2\vert\mathbf{y}_1])=\sigma_u^2\mathbf{G}_{21}\mathbf{TG}_{12}$ (S10.e)	
Cov. Predictions and realized values	$Cov(\mathbf{u}_2,E[\mathbf{u}_2\vert\mathbf{y}_1])=Cov(\mathbf{y}_2,E[\mathbf{y}_2\vert\mathbf{y}_1])=\sigma_u^2\mathbf{G}_{21}\mathbf{TG}_{12}$ (S10.e)	
Prediction Error Variances	$Var(\mathbf{u}_2-E[\mathbf{u}_2\vert\mathbf{y}_1])=\sigma_u^2[\mathbf{G}_{22}-\mathbf{G}_{21}\mathbf{TG}_{12}]$ (S.10c)	$Var(\mathbf{y}_2-E[\mathbf{y}_2\vert\mathbf{y}_1])=\sigma_u^2[\mathbf{G}_{22}-\mathbf{G}_{21}\mathbf{TG}_{12}]+\mathbf{I}_2\sigma_\varepsilon^2$ (S.10d)

The first element on the right hand side of (S.10d), is the variance-covariance matrix of prediction errors of genetic values, $Var(\mathbf{u}_2-E[\mathbf{u}_2|\mathbf{y}_1])=\sigma_u^2[\mathbf{G}_{22}-\mathbf{G}_{21}\mathbf{TG}_{12}]$; this is simply the prior variance-covariance matrix of genetic values, $\sigma_u^2\mathbf{G}_{22}$ minus a quadratic form, given by $\sigma_u^2\mathbf{G}_{21}\mathbf{TG}_{12}$, that quantifies reduction in uncertainty about genetic values of individuals in the TST data set attained by observing phenotypes of the individuals in the TRN data set. If individuals in TRN and TST data sets, are independent, i.e., when $\mathbf{G}_{21}=\mathbf{0}$, the (co)variance of prediction errors of genetic values equals the prior variance (i.e., there is no statistical learning). The second term in the right-hand-side of (S.10d), $\mathbf{I}_2\sigma_\varepsilon^2$, represents uncertainty about future phenotypes due to error terms; since these are uncorrelated with the phenotypes in the TRN data set there is no learning about this component.

The diagonal elements of expressions S.10c and S.10d give the PEV of individual genetic values and of individual phenotypes, respectively,

$$Var\left(u_{n+1} - E\left[u_{n+1} \middle| \mathbf{y}_1\right]\right) = \sigma_u^2 \left[G_{n+1,n+1} - \sum_{i=1}^{n} \sum_{j=1}^{n} G_{n+1,i} G_{n+1,j} T_{ij}\right] \tag{S.10e}$$

$$Var\left(y_{n+1} - E\left[y_{n+1} \middle| \mathbf{y}_1\right]\right) = \sigma_u^2 \left[G_{n+1,n+1} - \sum_{i=1}^{n} \sum_{j=1}^{n} G_{n+1,i} G_{n+1,j} T_{ij}\right] + \sigma_\varepsilon^2 \tag{S.10f}$$

Since **T** is positive definite $\sum_{i=1}^{n} \sum_{j=1}^{n} G_{n+1,i} G_{n+1,j} T \geq 0$ with equality holding only when all the genomic relationships between individual *n+1* and individuals in the TRN data set are equal to zero.

R-squared. The proportional reduction of variance of genetic values accounted for by predictions is given by the following R-squared (genetic values):

$$\begin{aligned} R_{n+1,u}^2 &= 1 - \frac{Var\left(u_{n+1} - E\left[u_{n+1} \middle| \mathbf{y}_1\right]\right)}{Var\left(u_{n+1}\right)} \\ &= 1 - \frac{\sigma_u^2 \left[G_{n+1,n+1} - \sum_{i=1}^{n} \sum_{j=1}^{n} G_{n+1,i} G_{n+1,j} T_{ij}\right]}{\sigma_u^2 G_{n+1,n+1}} \\ &= \frac{\sum_{i=1}^{n} \sum_{j=1}^{n} G_{n+1,i} G_{n+1,j} T_{ij}}{G_{n+1,n+1}} \end{aligned} \tag{S.11}$$

A similar R-squared for prediction of phenotypes is:

$$\begin{aligned} R_{n+1,y}^2 &= 1 - \frac{Var\left(y_{n+1} - E\left[y_{n+1} \middle| \mathbf{y}_1\right]\right)}{Var\left(y_{n+1}\right)} \\ &= 1 - \frac{\sigma_u^2 \left[G_{n+1,n+1} - \sum_{i=1}^{n} \sum_{j=1}^{n} G_{n+1,i} G_{n+1,j} T_{ij}\right] + \sigma_\varepsilon^2}{\sigma_u^2 G_{n+1,n+1} + \sigma_\varepsilon^2} \\ &= \frac{\sigma_u^2 \sum_{i=1}^{n} \sum_{j=1}^{n} G_{n+1,i} G_{n+1,j} T_{ij}}{\sigma_u^2 G_{n+1,n+1} + \sigma_\varepsilon^2} \end{aligned}$$

The ratio $R_{n+1,y}^2 / R_{n+1,u}^2 = \frac{\sigma_u^2 G_{n+1,n+1}}{\sigma_u^2 G_{n+1,n+1} + \sigma_\varepsilon^2} = h_{n+1}^2$ where h_{n+1}^2 is the heritability of the trait for individual *n+1* (note that, when $G_{n+1,n+1} = 1$, h_{n+1}^2 equals the heritability of the trait, $h^2 = \frac{\sigma_u^2}{\sigma_u^2 + \sigma_\varepsilon^2}$); therefore,

$$R^2_{n+1,y} = h^2_{n+1} R^2_{n+1,u} = h^2_{n+1} \frac{\sum_{i=1}^{n} \sum_{j=1}^{n} G_{n+1,i} G_{n+1,j} T_{ij}}{G_{n+1,n+1}} \qquad \text{(S.12)}$$

Suppose now that all the off-diagonal elements of $\mathbf{G}_{11}$ (i.e., all the G_{ij} such that $i, j \in (1,..., n)\, ; i \neq j$) are zero. In this case **T** is diagonal and the R-squared formula reduces to $R^2_{n+1,y} = h^2_{n+1} \frac{\sum_{i=1}^{n} G^2_{n+1,i}}{G_{n+1,n+1}}$. When data involve nominally unrelated individuals the off-diagonal elements of $\mathbf{G}_{11}$ are expected to be small, but not exactly equal to zero. Relative to the case where the TRN data set comprise independent TRN phenotypes, the use of correlated TRN phenotypes yields smaller R-squared. This happens because, other things being equal, the amount of information provided by the TRN data set comprises genetically independent individuals. Therefore, the index $\frac{h^2_{n+1}}{G_{n+1,n+1}} \sum_{i=1}^{n} G^2_{n+1,i}$ acts as an upper-bound to the R-squared; specifically,

Case 1 (perfect LD between markers and QTL): $$R^2_{n+1,y} \leq \frac{h^2_{n+1}}{G_{n+1,n+1}} \sum_{i=1}^{n} G^2_{n+1,i} \,. \qquad \text{(S.13)}$$

2.2. Case 2: when genomic relationships are computed using markers (i.e., imperfect LD between markers and QTL)

In practice, genomic relationships are computed from marker genotypes that are in imperfect LD with genotypes at causal loci. Further, since the patterns of realized genomic relationships at different sets of loci (e.g., markers and causal loci) vary across the genome [4], realized genomic relationships at markers ($\bar{G}_{n+1,i}$) should be regarded as proxies for the realized genomic relationships at causal loci ($G_{n+1,i}$).

When marker-derived genomic relationships are used in place of realized genomic relationships at causal loci, the assumptions of model (S.8) do not hold. Therefore it is not possible to derive closed-form expressions for prediction R-squared. This problem can be circumvented by deriving a closed form expression for an upper bound to prediction R-squared. To arrive at this closed form we assume that genomic relationships realized at causal loci among pairs of individuals in the TRN data set are known. Essentially, this has the effect of treating matrix **T** as a known constant. Therefore, we consider only the

impacts of imperfect LD between markers and QTL that occurs through misspecification of genomic relationships between individuals in the TST and those in the TRN data set. Predictions in G-BLUP are given by $\sum_{i=1}^{n} G_{n+1,i}\tilde{y}_i$. Because of the assumption that genomic relationships at causal loci among individuals in the TRN data set are known, inferences about the $\tilde{y}_i$'s, the entries of the vector $\tilde{\mathbf{y}} = [\mathbf{G} + \mathbf{I}\lambda]^{-1}\mathbf{y}$, are not affected by imperfect LD between markers and QTL.

Assume that the realized genomic relationships at markers between an individual in the TST data set (*n+1*) and all individuals in the TRN data set, $\{\overline{G}_{n+1,1},...,\overline{G}_{n+1,n}\}$, can be described using a linear regression on genomic relationships realized at causal loci, $\{G_{n+1,1},...,G_{n+1,n}\}$, that is

$$\overline{G}_{n+1,i} = b_{n+1}G_{n+1,i} + \xi_{n+1,i}\,, \qquad (i=1,...,n)$$

where b_{n+1}represents the regression of marker-derived genomic relationships $\{\overline{G}_{n+1,1},...,\overline{G}_{n+1,n}\}$ on realized genomic relationships at causal loci $\{G_{n+1,1},...,G_{n+1,n}\}$and $x_{n+1.i}$ represents an error term which accounts for differences in realized genomic relationships at markers and QTL. A similar approach was used before by Yang and co-authors [5]. However, the regression used by these author applied to all the entries of the relationship matrix, including diagonal and off-diagonal terms. In our case, we focus on quantifying the effects of imperfect LD that occur through miss-specification of TRN-TST relationships; therefore the regression is based on the off-diagonal terms only.

Using $\overline{G}_{n+1,i} = b_{n+1}G_{n+1,i} + \xi_{n+1,i}$ in (S.9b) we get:

$$\begin{aligned}\overline{E}[u_{n+1}|\mathbf{y}_1] &= \sum_{i=1}^{n}\overline{G}_{n+1,i}\sum_{j=1}^{n}T_{ij}y_j \\ &= \sum_{i=1}^{n}\overline{G}_{n+1,i}\tilde{y}_i \\ &= \sum_{i=1}^{n}(b_{n+1}G_{n+1,i} + \xi_{n+1,i})\tilde{y}_i\end{aligned}$$

The term $\xi_{n+1,i}$ is, by construction, orthogonal to the realized genomic relationships at causal loci; therefore, for large *n* it is safe to assume that $\sum_{i=1}^{n}\xi_{n+1,i}\tilde{y}_i$ approaches zero. Using $\sum_{i=1}^{n}\xi_{n+1,i}\tilde{y}_i = 0$ in the above expression, $\overline{E}[u_{n+1}|\mathbf{y}_1] = b_{n+1}\sum_{i=1}^{n}G_{n+1,i}\tilde{y}_i = b_{n+1}E[u_{n+1}|\mathbf{y}_1]$. Therefore, the variance of prediction errors is now given by

$$\begin{aligned} Var\left(u_{n+1}-\overline{E}\left[u_{n+1}|\mathbf{y}_1\right]\right) &= Var\left(u_{n+1}-b_{n+1}E\left[u_{n+1}|\mathbf{y}_1\right]\right) \\ &= Var\left(u_{n+1}\right)+b^2_{n+1}Var\left(E\left[u_{n+1}|\mathbf{y}_1\right]\right) - 2b_{n+1}Cov\left(u_{n+1},E\left[u_{n+1}|\mathbf{y}_1\right]\right) \end{aligned}$$

It can be shown (see expression (S10.e) in Table S1) that the variance of the conditional expectation, $Var\left(E\left[u_{n+1}|\mathbf{y}_1\right]\right)$, equals the covariance between the true genetic values and the BLUP, $Cov\left(u_{n+1},E\left[u_{n+1}|\mathbf{y}_1\right]\right)$. Specifically, from (S10.e) we have:

$$Var\left(E\left[u_{n+1}|\mathbf{y}_1\right]\right)=Var\left[u_{n+1}|\mathbf{y}_1\right]=Cov\left(u_{n+1},E\left[\mathbf{u}_2|\mathbf{y}_1\right]\right)=\sigma^2_u\mathbf{g}'_{\mathrm{n+1}}\mathbf{T}\mathbf{g}_{\mathrm{n+1}}=\sigma^2_u\sum_{i=1}^{n}\sum_{j=1}^{n}G_{n+1,i}G_{n+1,j}T_{ij}$$

, where $\mathbf{g}_{n+1}=\left\{G_{n+1,1},...,G_{n+1,n}\right\}'$ is a vector containing the genomic relationships realized at causal loci between individual *n+1* and every individual in the TRN data set. This is simply a scalar version of (S10.e). Therefore,

$$\begin{aligned} Var\left(u_{n+1}-\overline{E}\left[u_{n+1}|\mathbf{y}_1\right]\right) &= Var\left(u_{n+1}\right)+b^2_{n+1}Var\left(E\left[u_{n+1}|\mathbf{y}_1\right]\right) - 2b_{n+1}Cov\left(u_{n+1},E\left[u_{n+1}|\mathbf{y}_1\right]\right) \\ &= \sigma^2_{\mathrm{u}}\left\{\mathrm{G}_{\mathrm{n+1,n+1}}+\left(b^2_{n+1}-2b_{n+1}\right)\sum_{i=1}^{n}\sum_{j=1}^{n}G_{n+1,i}G_{n+1,j}T_{ij}\right\} \\ &= \sigma^2_{\mathrm{u}}\left\{\mathrm{G}_{\mathrm{n+1,n+1}}-\left(2b_{n+1}-b^2_{n+1}\right)\sum_{i=1}^{n}\sum_{j=1}^{n}G_{n+1,i}G_{n+1,j}T_{ij}\right\} \end{aligned}$$

Using $\left(2b_{n_1+1}-b^2_{n_1+1}\right)=1-(1-b_{n_1+1})^2$ the prediction R^2 becomes

$$\begin{aligned} \overline{R}^2_{n+1,u} &= 1-\frac{Var\left(u_{n+1}-\overline{E}\left[u_{n+1}|\mathbf{y}_1\right]\right)}{Var\left(u_{n+1}\right)} \\ &= 1-\frac{\sigma^2_u\left\{G_{n+1,n+1}-\left[1-(1-b_{n_1+1})^2\right]\sum_{i=1}^{n}\sum_{j=1}^{n}G_{n+1,i}G_{n+1,j}T_{ij}\right\}}{\sigma^2_u G_{n+1,n+1}} \\ &= \left[1-(1-b_{n_1+1})^2\right]\frac{\sum_{i=1}^{n}\sum_{j=1}^{n}G_{n+1,i}G_{n+1,j}T_{ij}}{G_{n+1,n+1}} \\ &= \left[1-(1-b_{n_1+1})^2\right]R^2_{n+1,u} \end{aligned}$$

And for prediction of phenotypes we have:

$$\overline{R}^2_{n+1,y}=h^2_{n+1}\left[1-(1-b_{n_1+1})^2\right]R^2_{n+1,u}=\left[1-(1-b_{n_1+1})^2\right]R^2_{n+1,y}$$

and, $\dfrac{R^2_{n+1,y}-\overline{R}^2_{n+1,y}}{R^2_{n+1,y}}=(1-b_{n_1+1})^2$.

Therefore, the coefficient $\tau_{n+1} = (1 - b_{n_1+1})^2$, can be regarded as a minimum shrinkage factor on R-squared due to use of marker-derived genomic relationships, as opposed to those realized at causal loci. This is a minimum shrinkage factor because in its derivation we have assumed that genomic relationships at causal loci were known for individuals at the TRN data set. Hence, we have

Case 2 (imperfect LD): $\bar{R}^2_{n+1,y} \le h^2_{n+1}\left[1-(1-b_{n_1+1})^2\right]R^2_{n+1,u}$ (S.14)

Under perfect LD and with infinite sample size, $R^2_{n+1,u}$ reaches one. Therefore, the term $h^2_{n+1}\left[1-(1-b_{n_1+1})^2\right]$ can be interpreted as an 'optimistic' upper bound on R-squared of predictions of phenotypes that be attained using G-BLUP type methods.

The term $\tau_{n+1} = (1 - b_{n_1+1})^2$ plays a central role in upper-bounds on R-squared. This coefficient has a maximum at $b_{n+1} = 1$, a case that would occur if markers and causal loci and in perfect LD. Values of b_{n+1} smaller or larger than one yield $\left[1-(1-b_{n_1+1})^2\right] < 1$, reducing the maximum R-squared that can be attained.

In practice, the set of causal loci is unknown; however, an approximation to b_{n+1} can be obtained by computing realized genomic relationships, $\{\bar{G}_{n+1,1},...,\bar{G}_{n+1,n}\}$ and $\{G_{n+1,1},...,G_{n+1,n}\}$, at disjoints sets of loci and regressing $\{\bar{G}_{n+1,1},...,\bar{G}_{n+1,n}\}$ on $\{G_{n+1,1},...,G_{n+1,n}\}$. We present estimates of these coefficients in the article and discuss the impact of linkage on this regression by comparing estimates of these regressions derived using data with related and unrelated individuals.

1. Bayesian Implementation

The model described by expression S.4 was implemented with two modifications: (a) the model was extended by inclusion of an intercept, and (b) variance parameters were treated as unknown and were estimated from the training data sets. The model was implemented in a Bayesian setting; this requires assigning prior distributions to the unknown intercept and variance components. The intercept was assigned a flat prior and variance components were assigned independent Scaled-Inverse Chi-square densities. Therefore, the joint posterior density of the unknowns was

$$p(\mu,\mathbf{u},\sigma_u^2,\sigma_\varepsilon^2|\mathbf{y},df,S_\varepsilon,S_u) \propto p(\mathbf{y}|\mathbf{u},\sigma_\varepsilon^2)p(\mathbf{u}|\sigma_u^2)p(\sigma_u^2,\sigma_\varepsilon^2)$$
$$\propto \mathrm{MVN}(\mathbf{y}|\mathbf{1}\mu+\mathbf{u},\mathbf{I}\sigma_\varepsilon^2)\mathrm{MVN}(\mathbf{u}|\mathbf{0},\mathbf{G}\sigma_u^2)\chi^{-2}(\sigma_\varepsilon^2|df,S_\varepsilon)\chi^{-2}(\sigma_u^2|df,S_u) \quad \text{(S.15)}$$

where, $\chi^{-2}(\sigma_{\cdot}^2|df,S_{\cdot})$ denotes an Inverted-Scaled Chi-Squared density for the random variable $\sigma_{\cdot}^2$ and with degree of freedom and scale parameters *df* and $S_{\cdot}$, respectively. The degree of freedom parameter was set equal to 5 and the scale was chosen so that the prior expected values of the variance parameters equals the phenotypic variance (1 in the simulated data and approximately 40 in the real data) times 0.8 for the genomic variance and times 0.2 in case of the residual variance.

Samples from the posterior density were obtained with a Gibbs sampler that uses an orthogonal representation of the model (i.e., a random regression on the eigenvectors of **G**). The algorithm is fully described in [6,7]. A total of 18,000 samples per run were drawn. Of these, the first 3,000 were discarded as burn-in. The remaining 15,000 samples were thinned with a thinning interval of 3, yielding total of 5,000 samples to compute Monte Carlo estimates of the unknowns. The software used to carry out analyses is available upon request to the corresponding author.

Literature Cited

1. Henderson CR (1975) Best linear unbiased estimation and prediction under a selection model. Biometrics 31: 423–447.
2. Pszczola M, Strabel T, Mulder HA, Calus MPL (2012) Reliability of direct genomic values for animals with different relationships within and to the reference population. Journal of dairy science 95: 389–400.
3. Hoerl AE, Kennard RW (1970) Ridge regression: Biased estimation for nonorthogonal problems. Technometrics 12: 55–67.
4. Hill WG, Weir BS (2011) Variation in actual relationship as a consequence of Mendelian sampling and linkage. Genetics Research 93: 47–64. doi:10.1017/S0016672310000480.
5. Yang J, Benyamin B, McEvoy BP, Gordon S, Henders AK, et al. (2010) Common SNPs explain a large proportion of the heritability for human height. Nature genetics 42: 565–569.
6. de los Campos G, Gianola D, Rosa GJM, Weigel KA, Crossa J (2010) Semi-parametric genomic-enabled prediction of genetic values using reproducing kernel Hilbert spaces methods. Genetics Research 92: 295–308.
7. Janss L, de los Campos G, Sheehan N, Sorensen DA (2012) Inferences from Genomic Models in Stratified Populations. GENETICS: 693–704. doi:10.1534/genetics.112.141143.

1. Henderson CR (1975) Best linear unbiased estimation and prediction under a selection model. Biometrics 31: 423–447.

2. Pszczola M, Strabel T, Mulder HA, Calus MPL (2012) Reliability of direct genomic values for animals with different relationships within and to the reference population. Journal of dairy science 95: 389–400.

3. Hoerl AE, Kennard RW (1970) Ridge regression: Biased estimation for nonorthogonal problems. Technometrics 12: 55–67.

4. Hill WG, Weir BS (2011) Variation in actual relationship as a consequence of Mendelian sampling and linkage. Genetics Research 93: 47–64. doi:10.1017/S0016672310000480.

5. Yang J, Benyamin B, McEvoy BP, Gordon S, Henders AK, et al. (2010) Common SNPs explain a large proportion of the heritability for human height. Nature genetics 42: 565–569.

6. De los Campos G, Gianola D, Rosa GJM, Weigel KA, Crossa J (2010) Semi-parametric genomic-enabled prediction of genetic values using reproducing kernel Hilbert spaces methods. Genetics Research 92: 295–308.

7. Janss L, de los Campos G, Sheehan N, Sorensen DA (2012) Inferences from Genomic Models in Stratifi_ed Populations. GENETICS: 693–704. doi:10.1534/genetics.112.141143.
